# Technical Advances of the Recombinant Antibody Microarray Technology Platform for Clinical Immunoproteomics

**DOI:** 10.1371/journal.pone.0159138

**Published:** 2016-07-14

**Authors:** Payam Delfani, Linda Dexlin Mellby, Malin Nordström, Andreas Holmér, Mattias Ohlsson, Carl A. K. Borrebaeck, Christer Wingren

**Affiliations:** 1 Department of Immunotechnology and CREATE Health, Lund University, Medicon Village, Lund, Sweden; 2 Immunovia AB, Lund, Sweden; 3 Computational Biology & Biological Physics, Department of Astronomy and Theoretical Physics, Lund University, Lund, Sweden; National Central University, TAIWAN

## Abstract

In the quest for deciphering disease-associated biomarkers, high-performing tools for multiplexed protein expression profiling of crude clinical samples will be crucial. Affinity proteomics, mainly represented by antibody-based microarrays, have during recent years been established as a proteomic tool providing unique opportunities for parallelized protein expression profiling. But despite the progress, several main technical features and assay procedures remains to be (fully) resolved. Among these issues, the handling of protein microarray data, i.e. the biostatistics parts, is one of the key features to solve. In this study, we have therefore further optimized, validated, and standardized our in-house designed recombinant antibody microarray technology platform. To this end, we addressed the main remaining technical issues (e.g. antibody quality, array production, sample labelling, and selected assay conditions) and most importantly key biostatistics subjects (e.g. array data pre-processing and biomarker panel condensation). This represents one of the first antibody array studies in which these key biostatistics subjects have been studied in detail. Here, we thus present the next generation of the recombinant antibody microarray technology platform designed for clinical immunoproteomics.

## Introduction

High-performing tools for multiplexed protein expression profiling of minimal amounts of crude clinical samples will be essential in the quest for deciphering disease-associated biomarkers for e.g. diagnosis and prognosis [1–3]. A useful technology platform should be able to decode complex biological samples into detailed protein maps, as well as to filter and interpret these big data sets in terms of candidate biomarkers. The latter should result in both a full list of markers, reflecting the disease biology, and a condensed panel of biomarkers, displaying the best discriminatory power for e.g. diagnosis. This will, however, place high demands on the performance of the selected technology.

During the last several years, affinity proteomics, represented mainly by antibody microarrays [4–7], have been developed and established as a key tool within proteomics, providing opportunities for parallelized protein expression profiling, for review see [2, 8–10]. The platforms have been successfully used for delineating low- to high-abundant serum, plasma, urine, and/or tissue biomarkers associated with various forms of cancers and autoimmune disorders, see e.g. [5, 6, 11, 12]. But despite the progress, a number of key technical features (e.g. quality controls, specificity, functionality, and/or reproducibility) and key procedures (e.g. protein array data handling, i.e. the biostatistics part) remains to be validated, standardized, and implemented [8, 9]. In particular, the biostatistics of protein microarrays represents one of the key central steps that has not yet been adequately addressed.

**Fig 1:**
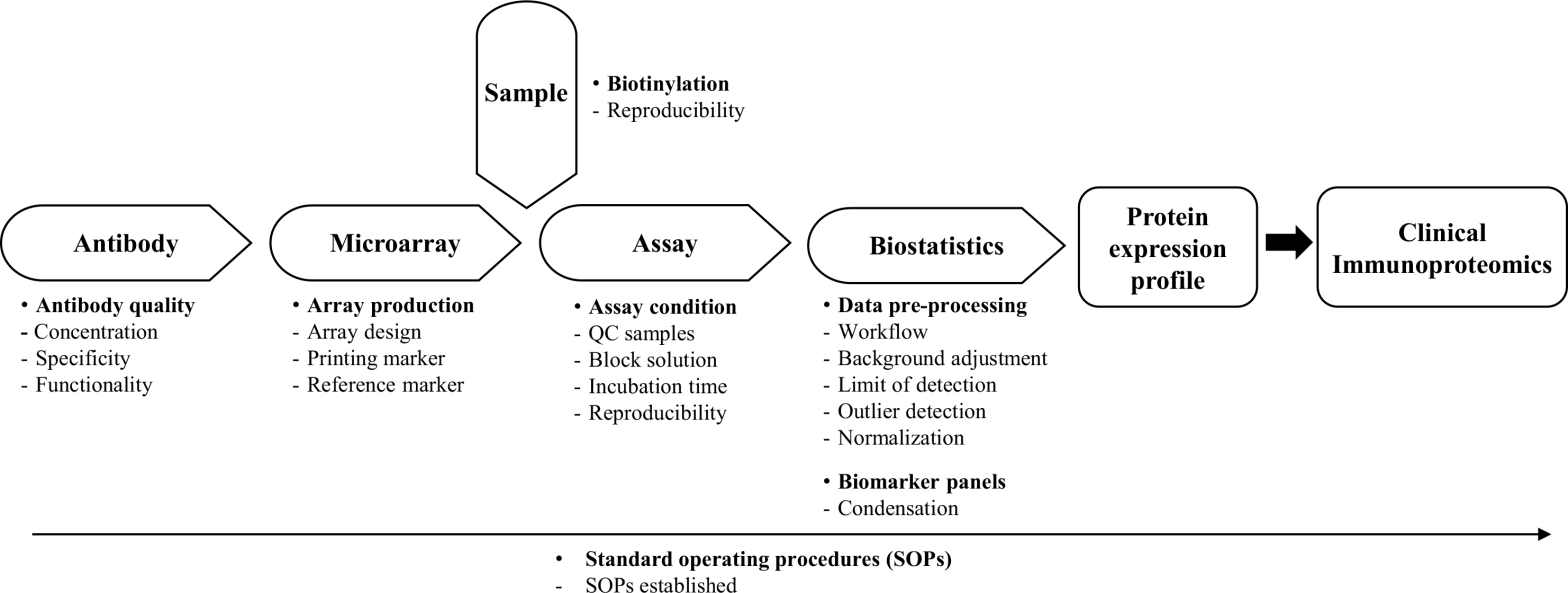
The key technological features involved in the design of our recombinant antibody microarray technology platform, outlining the specific, individual features uniquely addressed in this study.

The process of designing, developing and applying high-performing antibody microarrays for clinical proteomics is a complex process and requires a truly cross-disciplinary approach to be adopted [9]. To this end, five key methodology areas must be addressed in a parallel manner, including i) antibody design, ii) microarray design, iii) sample handling, iv) microarray assay, and v) biostatistics. Adopting this strategy, we have during the last decade developed and established a recombinant antibody microarray technology platform for clinical immunoproteomics [9, 13, 14]. The latter means that we explore the immune system as an early and specific sensor for disease by targeting mainly immunoregulatory proteins. In this study, we have further optimized, validated, and standardized our in-house designed technology platform [4, 7, 11] by addressing the main remaining technical features (e.g. antibody quality, array production, biotinylation, and selected assay conditions) and most importantly the biostatistics part (e.g. array data pre-processing and biomarker panel condensation) (see Fig 1). Here, we thus present the next generation of our recombinant antibody microarray technology platform designed for clinical immunoproteomics.

## Material and Methods

### Standard Operating Procedures

Standard operating procedure protocols (SOPs) were generated for each step, ranging from sample handling to microarray data analysis, resulting in a standardized SOP for running the recombinant scFv antibody microarray technology platform for clinical immunoproteomics.

### Samples

We used three cohorts of de-identified crude serum samples (marked healthy or non-healthy/diseased), denoted cohort 1 to 3, collected at Skåne University Hospital (Lund, Sweden). No clinical information or patient identifiers were retained for samples (since this information was neither needed nor used in this study). The work was approved by the regional ethics review board in Lund, Sweden (LU378-02, LU608-00, LU-30-03, LU513-01). Written consent was taken from participants. The samples were aliquoted and stored at -20°C until use. In serum sample cohort 1, 50 samples were mixed to create a reference serum sample, while the others were handled as individual samples, marked as either diseased (n = 151) or healthy (n = 57). Serum sample cohort 2 was composed of 341 samples, marked as either diseased (n = 171) or healthy (n = 170). Serum sample cohort 3 was composed of 1331 samples, marked as either diseased (n = 443) or healthy (n = 888).

### Quality control samples

Three types of standardized quality control (QC) serum samples, denoted QC_ref_, QC_label_ and QC_norm_, were introduced. QC_ref_ is based on pooled human serum samples (SeraCare Life Sciences, Milford, MA, USA), which is biotinylated in one batch (see below) and stored as aliquots, and then processed in one subarray on each microarray slide. QC_ref_ is intended to be used for evaluation of systematic technical variations and, if so required, for evaluation of the performance of the selected microarray data normalization procedure. QC_label_ is based on the same pooled human serum samples (SeraCare Life Sciences, Milford, MA, USA), but in contrast to QC_ref_, it is biotinylated alongside the target samples of the study at hand. QC_label_ is intended to be used as a control for the biotinylation step. QC_norm_ is prepared based on combining aliquots of equal volume of serum samples from each study group (e.g. healthy and diseased groups) from the study at hand. QC_norm_ is intended to be used as an alternative route for microarray data normalization if so required.

### Labeling of serum samples

The crude serum samples were labeled with EZ-link Sulfo-NHS-LC-Biotin (Pierce, Rockford, IL, USA) using a recently optimized labeling protocol for serum proteomes [4, 7]. Briefly, the samples were diluted 1:45 in PBS (~2mg protein/ml), and biotinylated at a molar ratio of biotin:protein of 15:1. Unreacted biotin was removed by extensive dialysis against PBS (pH 7.4) for 72 h at 4°C. The samples were aliquoted and stored at -20°C until further use.

### Production and purification of antibodies

In total, 377 antibodies, comprising of i) two monoclonal antibodies against CA-19-9 (Nordic BioSite, Stockholm, Sweden), and 375 human recombinant single-chain variable fragment (scFv) antibodies, including 356 antibodies targeting mainly immunoregulatory proteins and 19 scFv antibodies targeting short amino acid motifs (4 to 6 amino acids long) [15] were selected from a large phage display library [16] (Säll *et al*, manuscript submitted) (S1 Table). The specificity, affinity (normally in the nM range), and on-chip functionality of these phage display derived scFv antibodies were ensured by using i) stringent phage-display selection and screening protocols (using different sample formats, ranging from pure proteins and mixtures of pure proteins to crude samples) [16], ii) multiple clones (1 to 9) per protein, and iii) a molecular design, adapted for microarray applications [14]. In addition, the specificity of several of the antibodies have been validated using pure proteins, mixtures of pure proteins, as well as well-characterized, standardized serum samples (with i) known levels of the targeted analytes, ii) spiked with known level of specific protein(s) and/or iii) specific protein(s) depleted), and/or orthogonal methods, such as mass spectrometry (affinity pull-down experiments), ELISA, MesoScaleDiscovery assay, and cytometric bead assay, as well as using blocking experiments (S1 Table) [4, 7, 11, 17–21]

All scFv antibodies were produced in *E*. *coli* and purified from either i) expression supernatants using affinity chromatography on Ni^2+^-NTA agarose (Qiagen, Hilden, Germany), or ii) periplasmic space with the use of MagneHis^TM^ protein purification system (Promega Corporation, Madison, WI, USA) and a KingFisher Flex robot (Thermo Fisher Scientific, Waltham, MA, USA). The elution buffer (250 mM Imidazole) was exchanged for PBS through either extensive dialysis (supernatants) or using Zeba 96-well desalt spin plates (Thermo Fisher Scientific). The protein concentration was determined by measuring the absorbance at 280nm using NanoDrop-1000 (Thermo Acientific, Wilmington, DE, USA). The degree of purity and integrity of the scFv antibodies were evaluated by 10% SDS-PAGE (Invitrogen, Carlsbad, CA, USA). All antibodies were stored at 4°C until use.

### Production of antibody microarrays

Thirty μL scFv antibody was added per well to the 384-well printing source plate (black polypropylene, NUNC A/S, Roskilde, Denmark), containing 3 μL 1 μg/ml Alexa Fluor 555-Cadaverine (Thermo Fisher Scientific, Waltham, MA, USA). Fluorophore-labeled cadaverine was used as a spotting control and, if so required, for assisting in the spot finding step during array quantification. The antibodies were printed on black polymer MaxiSorp microarray slides (NUNC), by spotting one drop (~330 pl) at each position, using a non-contact printer (SciFlexarrayer S11, Scienion, Berlin, Germany). Four different microarray layouts were used, denoted array layout A to D, including a 94-plex antibody array (25x25 spots) (column x row), a 184-plex antibody array (32x22 spots), 195-plex antibody array (25x28 spots), and 351-plex antibody array (36x34 spots).

In layout A, 25x25 subarrays were printed, composed of 94 scFv antibodies, one negative control (PBS), and one positive control/reference marker (biotinylated BSA (b-BSA)). Ten 25x25 subarrays per slide were printed. Within each individual subarray, each scFv antibody was printed in 5 subsequent replicates, while PBS was spotted in 5 subsequent replicates at six locations spread across the subarray (i.e. in total 30 replicates). Further, b-BSA was printed as five full rows (5x25 spots), and spread across the subarray, and used to control for any surface defects. In total, nine slides were produced in three individual spot runs (three slides/day). Based on inadequate printing quality, only 83 scFv antibodies were used in the subsequent data analysis steps.

In layout B, 32x22 subarrays were printed, composed of 184 scFv antibodies, one negative control, and one positive reference marker. Each subarray was divided in three identical segments where a row of b-BSA consisting of 32 replicate spots was printed at the beginning and the end of each segment, i.e. four lines. Each scFv antibody was dispensed in triplicates, one in each segment, to assure adequate reproducibility. The negative control was printed in eight replicates per segment. Thirteen subarrays per slide were printed, and in total six slides were generated. Due to printing issues, only the first five rows in each segment, corresponding to 121 antibodies, were used in the subsequent data analysis.

In layout C, 25x28 subarrays were printed, composed of 195 scFv antibodies, one negative control, and one positive reference marker. The subarrays were divided and printed in three identical segments as in layout B, but with the following changes; the negative control was printed in five replicates per segment and b-BSA was printed in 25 replicates. Fourteen subarrays per slide were printed. In total, sixteen slides were produced in two individual spot runs (eight slides/day).

In layout D, 36x34 subarrays were printed, composed of 351 antibodies, one negative control, and one positive reference marker. The subarrays were divided and printed in three identical segments as in layout B, but with the following change; the negative control was printed in nine replicates per segment, and b-BSA was printed in 36 replicates. Fourteen subarrays were printed per slide.

### Analysis of antibody microarrays

Briefly, the printed microarray slides were allowed to dry for 2h (cohort I) or 1 week (cohorts II and III) at RT and were then mounted into a multi-well incubation chambers (NEXTERION® IC-16) (Schott, Jena, Germany). Next, the slides were blocked with 1% (v/v) Tween-20 (Merck Millipore) and 1% (w/v) fat-free milk powder (Semper, Sundbyberg, Sweden) in PBS (MT-PBS solution) for 2h (cohort I) or 1h (cohorts II and III) at RT. Of note, the composition of the original blocking solution (5% (v/v) fat-free milk in PBS) [22] was re-optimized (various combinations of Tween-20 and fat-free milk was tested) in order to reduce non-specific background binding and increase signal to noise ratios (data not shown). Subsequently, the slides were washed four times with 150 μl 0.05% (v/v) Tween-20 in PBS (T-PBS solution), and then incubated with 100 μl biotinylated serum sample, diluted 1:10 in MT-PBS solution (corresponding to a total serum dilution of 1:450), for 2h at RT under gentle agitation using an orbital shaker. Of note, the original sample incubation time (1h) [22) was re-optimized in order to increase signal intensities and signal to noise ratios (data not shown). After another washing step, the slides were incubated with 100 μl 1μg/ml Alexa 647-labelled streptavidin (SA647) (Invitrogen) in MT-PBS for 1h at RT under agitation. Finally, the slides were washed in T-PBS, and dried under a stream of nitrogen gas, and immediately scanned with a confocal microarray scanner (ScanArray Express, PerkinElmer Life & Analytical Sciences) at 10 μm resolution, using fixed scanner settings of 60% PMT gain and 90% laser power. The raw array data is available from the communicating author upon request.

### Data pre-processing

The ScanArray Express software v4.0 (PerkinElmer Life & Analytical Sciences) was used to quantify spot signal intensities, using the fixed circle method. Signal intensities with local background subtraction were used for data analysis. In the case of antibodies, each data point represents the mean value of all three replicate spots, unless any replicate CV exceeded 15%, in which case the worst performing replicate was eliminated and the average value of the two remaining replicates was used instead. Log2 values of signal intensities were used for subsequent analysis. The mean value was determined for the positive and negative control spots, using all data points.

In order to only include antibodies with a detectable signal, a limit of detection (LOD) cut-off was implemented. The cut-off was defined as mean PBS intensity plus 2xSD_PBS_. Antibodies that were found to have a mean signal intensity below LOD in > 70% of all analyzed samples were removed from the raw data set.

To identify technical outlier samples in the raw data, a two-step procedure was implemented. First, using the positive control (see above), the sample was excluded if the CV-value was ≥20% for at least 3 of 4 rows (also a way of identifying segments within a subarray with potential printing issues, see above). A visual inspection was conducted if 2 of 4 rows displayed inadequate CV-values, potentially leading to the use of only 1 or 2 segments of the subarray (made up of 3 identical segments). Second, a principle component analysis (PCA) was performed using the Qlucore Omics Explorer 3.1 software (Qlucore AB, Lund, Sweden) for visual identification of potential outliers.

### Standard data normalization

A two-step normalization strategy was used. In the first step, any differences between days (rounds) of analysis were normalized using the “subtract by group mean” approach [23]. In this approach, the mean value ($\bar{x}$) of each analyte (i) within each day of analysis was calculated (= ${\bar{x}}_{i}$), and subtracted from the respective individual values (x_i_), thus zero centering the data (= $x_{i}\text{-}{\bar{x}}_{i}$). In order to avoid negative and/or zero values in the data, the grand mean signal of each antibody over all days of analysis was calculated using the raw data, and was added to each respective data point. In the second step, any array-to-array differences were normalized using a modified semi-global normalization method, as previously described [11, 24]. In this approach, the standard deviation (SD) was first calculated for each antibody, and the twenty percent of the antibodies (X) displaying the lowest SD-values in all the subarrays were identified and used to calculate a normalization factor for each subarray: The normalization factor *N*_i_ was calculated by the formula *N*_i_ = *S*_i_/μ, where *S*_i_ was the sum of the log2 transformed signal intensities for the X analytes for each subarray and μ was the sum of the log2 transformed signal intensities for the X analytes averaged over all subarrays. Finally, the signal of each subarray was divided by the normalization factor *N*_i_.

### Evaluation of normalization strategies

In total, nine different normalization strategies, including our currently adopted normalization method, “subtract by group mean” strategy combined with “semi-global normalization” [11, 24], were tested and compared (Table 1). All approaches were also compared to un-normalized log2 transformed raw data to investigate the overall impact of the normalization process on a data set. At this stage, QC_norm_ was not explored as an alternative route for microarray data normalization.

**Table 1:** List of normalization processes evaluated in this study.

Un-normalized log2 transformed data
Global VSN
Global LOESS
Local LOESS
Local VSN
Quantile
Subtract by group mean + Semi-global
ComBat + Semi-global
Global VSN + Combat
Global LOESS + Combat

The different strategies included variance stabilization and normalization (VSN, vsn package) [25], LOESS (limma package) [26], quantile (preprocessCore package) [27] and ComBat (sva package) [28] methods in R using Bioconductor packages [29] (Table 1). LOESS and VSN normalizations were implemented globally (using all samples analyzed across all days of analysis) as well as locally (using all samples within each single day of analysis). In the case of LOESS normalization, the default parameters in limma package was used. When performing VSN normalization, the quantile that was used for the resistant least trimmed sum of squares regression, for the estimation of parameters, was set to 0.5 to ensure the robustness of the procedure. For quantile normalization, the default parameters in preprocessCore package was adopted. When applying ComBat normalization, a parametric prior method was selected, with the batches corresponding to days of analysis. In addition to the above-mentioned methods, a modified semi-global normalization method was used for linear scaling of data. Further, global LOESS and global VSN were also tested in combination with ComBat in an attempt to adjust for day-to-day variation. Finally, ComBat was also evaluated in combination with the semi-global normalization approach to adjust for array-to-array variation. For comparative evaluation of the different approaches, several qualitative measures, such as normal quantile-quantile plots, boxplots, density plots and meanSdPlots were utilized in R. Visualization of the samples by principal component analysis (PCA) and two-group comparisons (t-tests) were conducted in Qlucore Omics Explorer 3.1 software (Qlucore AB, Lund, Sweden).

In addition, supervised classification of samples was used when comparing different days of analysis. To this end, the Random Forest (RF) function implemented in the randomForest R package was also used to create a RF model with 1000 decision trees (ntree = 1000) [30, 31].

### Evaluation of methods for deciphering condensed biomarker signatures

Four different methods for defining a condensed biomarker signature providing the best classification of two sample groups were evaluated, including i) selection based on p-values, ii) backward elimination using support vector machine (SVM), iii) modified backward elimination using SVM consensus approach (SVMc), and iv) RF [32].

First, the biomarkers were ranked based on their Wilcoxon p-values, and the markers with the lowest p-values were selected.

In the case of backward elimination using an SVM (BE-SVM), an SVM with a linear kernel and soft margin parameter *C* = 1, was used as the classifier in a backward elimination scheme. Given a panel of all biomarkers available, a leave-one-out (LOO) cross-validation estimate of the AUC was calculated. Next, a ranking of the included biomarkers was established using all of the SVMs trained in the LOO cross-validation procedure [24]. The biomarker with the lowest ranking was removed from the panel and the procedure restarted by obtaining a new LOO cross-validation AUC estimate and a new ranking of the remaining biomarkers. This procedure was terminated when only one biomarker was left in the panel. This backward elimination scheme resulted in a plot of AUC (LOO cross-validation estimate) as a function of panel size together with a final biomarker ranking list.

Next, we evaluated a modified version of backward elimination using an SVMc approach. A potential problem with the above approach might be overtraining with respect to a given data cohort, especially when sample sizes are small. Randomly correlated biomarkers might obtain a high rank in the above procedure. To reduce the effect of such a potential overtraining, an additional *K*-fold cross-validation loop was added in which one of the *K*:th parts was removed from the data cohort before the initiation of the backward elimination scheme. The outermost was iterated, leaving out *K* parts of the data, hence resulting in *K* final ranking lists, possibly *N*x*K* lists if the outermost loop was randomly repeated *N* times. A consensus approach [24] was then used to combine these lists into a final biomarker ranking list, now with fewer random correlating biomarkers. In this study, we used K = 5 and N = 3, except for sample cohort 3, where N = 1 was used.

Both the above approaches are based on linear classifiers and might for some diagnostic problems lack the necessary complexity to reach high accuracy. SVM classification models could use non-linear kernels to allow for more complex classifiers, but with the cost of tuning more parameters. Random Forest (RF) models [32] are thus an interesting alternative, providing both non-linear capabilities and built-in feature ranking methods, with very few tuning parameters. Again to reduce overtraining with respect to specific data cohorts, an outer (repeated) K-fold cross-validation loop is used to obtain a final ranking list. This feature selection follows the procedure outlined in the caret package [33].

All condensed biomarker signatures, obtained by the different rankings presented above, were evaluated using a linear SVM. In those cases where the biomarker list was based on RF models, an evaluation using a RF was also added. The reason for this was that if non-linear effects was discovered by the RF ranking method, the use of a linear SVM during evaluation was not optimal. Thus, it was natural to also evaluate a RF ranking list by RF models.

## Results

In this study, we have further optimized, validated, and standardized our in-house designed recombinant antibody microarray technology platform for clinical immunoproteomics. This was accomplished by resolving the main technical features and assay procedures, and most importantly the protein microarray biostatistics step as specified in Fig 1. Collectively, these advances enabled us to formalize the entire platform and generate standard operating procedures (SOPs) for all steps of the set-up.

### Antibody quality control

We used recombinant scFv antibodies, selected from a large phage display library based on a molecular design adapted for microarray applications, as content and evaluated the probe quality in terms of spotting concentration, on-chip functionality and specificity.

**Fig 2:**
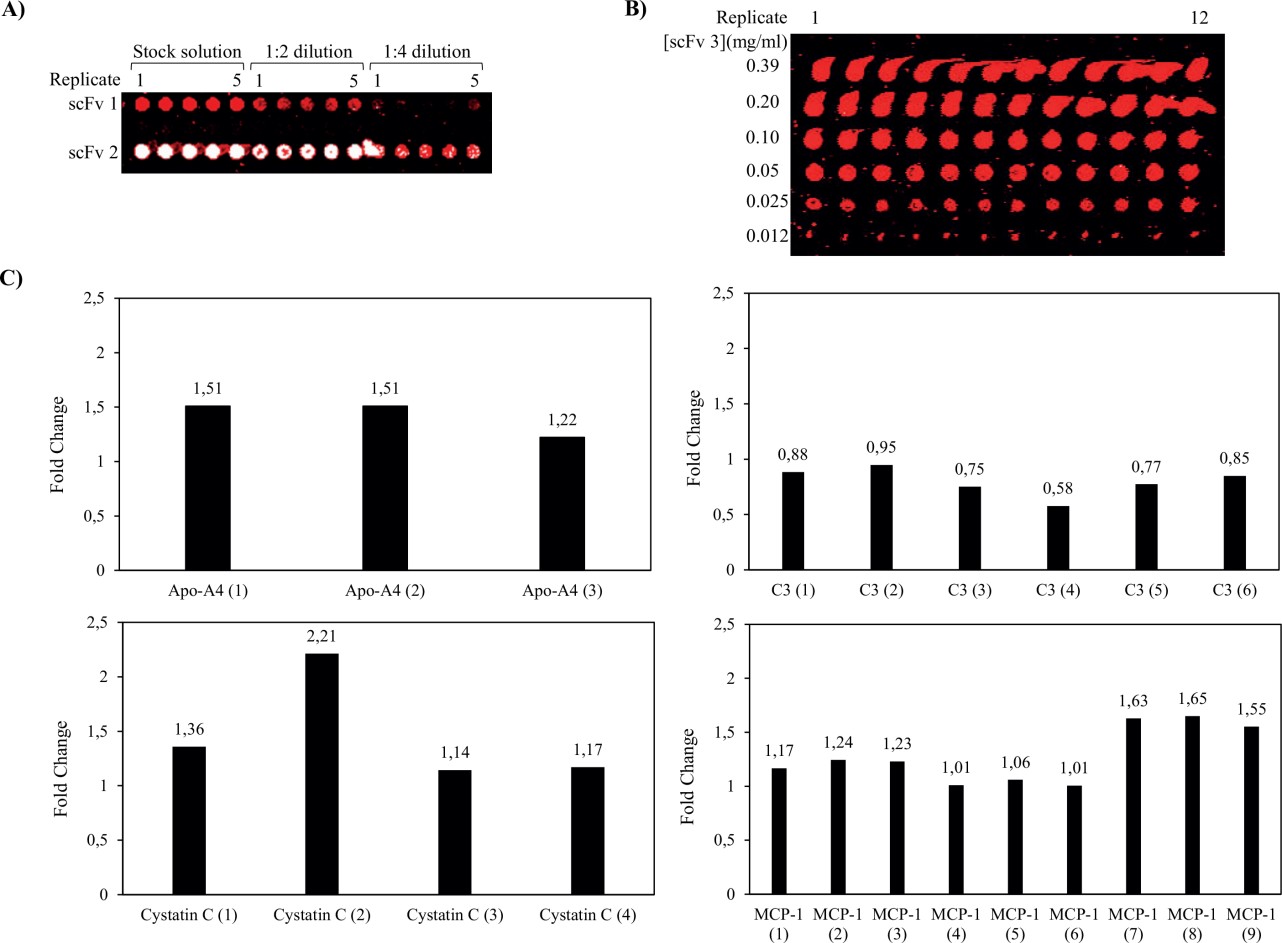
Evaluation of the antibody quality in terms of spotting concentration, on-chip functionality and specificity. (**A**) Two different scFvs antibodies, denoted as scFv1 (against complement protein C5) and scFv2 (against Myomesin 2), were dispensed in quintuplicate at three different concentrations, stock solution, 1:2 dilution and 1:4 dilution. The microarray was processed using a biotinylated serum sample. A scanner setting of 10 μm resolution, using 70% PMT gain and 90% laser power was used. (**B**) ScFv3 (anti-C1q) was spotted in 6 different concentrations, each in 12 replicate spots. The microarray was processed using pure antigen (5 nM pure Alexa 647-labeled C1q) and scanned at 10 μm resolution, using 60% PMT gain and 90% laser power. (**C**) Protein expression profiling of biotinylated serum sample. Several antibody clones directed towards the same antigen but against different epitopes were used, targeting Apolipoprotein-A4 (Apo-A4, n = 3), Complement factor 3 (C3, n = 6), Cystatin C (n = 4) and Monocyte Chemoattractant Protein-1 (MCP-1, n = 9). The observed signal intensities are given, in terms of fold change, in diseased vs. healthy samples.

First, we optimized and standardized the spotting concentration for each individual antibody. Representative results for three antibody clones, two targeting a crude biotinylated serum sample (Fig 2A) and one targeting a pure labelled protein (Fig 2B), are shown. Although dispensing of more concentrated antibody reagents generated 130 μm sized spots fully saturated with antibody probes (as viewed from the observed signal intensities), any antibody excess appeared to result in adverse smearing and tailing effects rather than continuously growing spot sizes. These poor spot features would significantly impair the subsequent quantification step. Further, the cut-off concentration for the observed effects appeared to be clone dependent, indicating that the antibodies populated (adsorbed to) the surface in a clone dependent manner. On the other hand, reducing the antibody spot concentration below the cut-off, resulted in smaller and smaller spots, indicating that the spots grew from the spot center and outwards. Hence, it is essential that the spotting concentration is optimized and standardized for each individual antibody (50–300 μg/ml) for a given set-up (e.g. dependent on choice of spotting buffer and solid support) in order to optimize the spot features (homogeneity) as well as to enable a comparison across different studies over time.

Stringent phage-display selection and screening protocols were used to promote antibody functionality and specificity (see Material and Methods). All the arrayed antibodies gave a detectable dynamic signal intensity (although sample dependent) when targeting crude, biotinylated serum samples (data not shown). Hence, the data indicated high on-chip functionality.

In order to further support the specificity claims, based on the selection criteria, orthogonal methods and on-chip confirmations (see Material and Methods for details), the microarrays were designed with a built-in specificity control step. To this end, we have included several antibody clones (n = 1 to 9) directed against the same antigen, but targeting different epitopes (the antibodies differ with respect to the amino acid sequence of their complementarity determining regions). Representative data for three to nine antibody clones directed against four different protein antigens, including apolipoprotein-A4, complement factor 3 (C3), Cystatin C and monocyte chemoattractant protein-1 (MCP-1), are shown in Fig 2C. In each case, the data showed that the antibodies gave similar patterns with respect to up- vs. down-regulations when comparing diseased vs. healthy controls. Although the observed fold changes per antibody were moderate (a common feature for antibody arrays targeting moderate to low abundant targets), they were highly reproducible. Hence, the data further supported the on-chip specificity and functionality of the arrayed antibodies through this built-in assay feature at large.

### Microarray quality control

**Fig 3:**
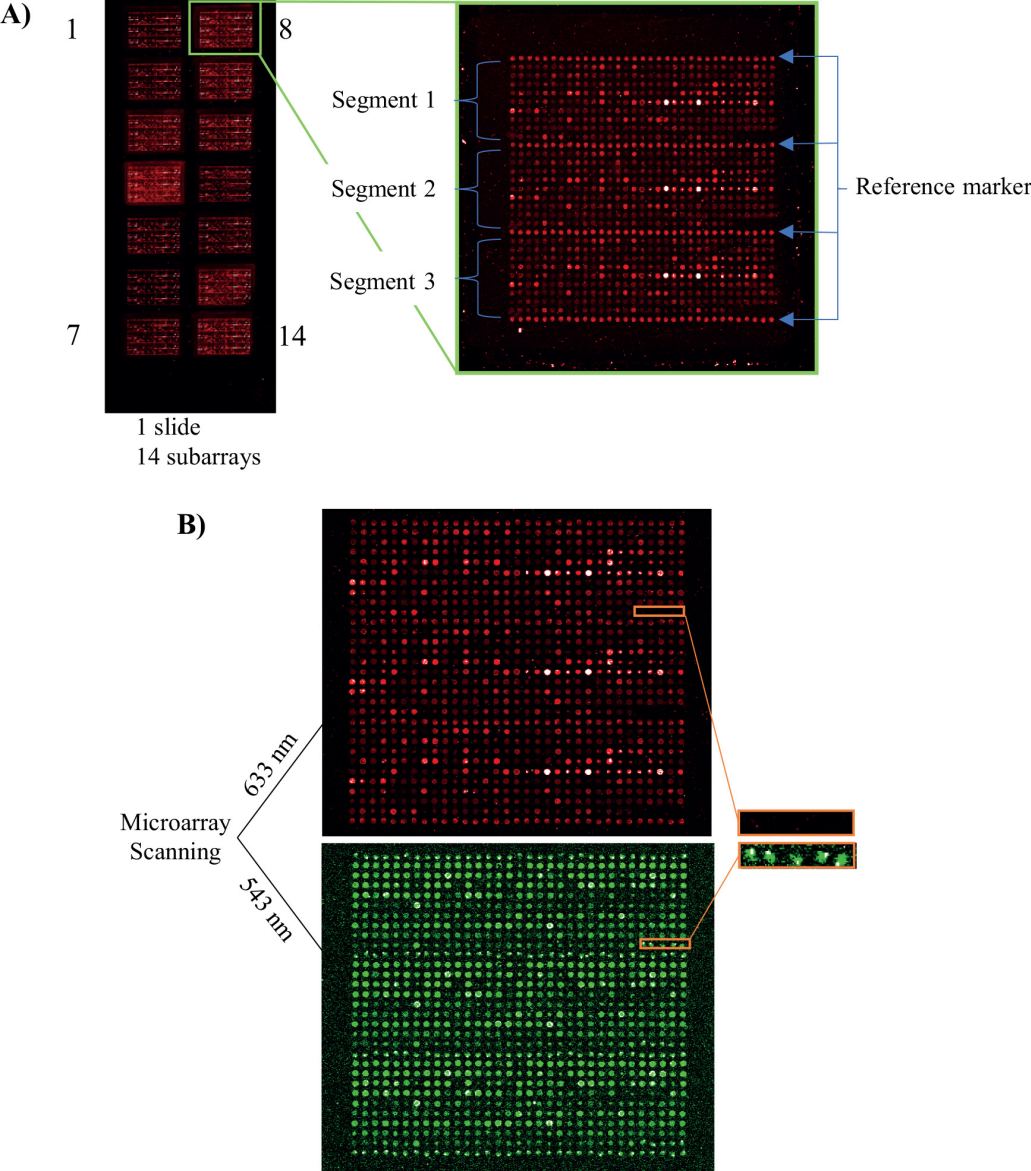
Evaluation of microarray quality in terms of array design and printing markers. (**A**) Representative image of a microarray slide with 14 identical subarrays. Each subarray (n = 14) is divided into 3 segments separated by dispensed rows of reference marker replicate spots (n = 4). (**B**) Blank replicate spots (n = 5) detected in the marked area. No signal intensity was obtained from the dispensed blank replicate spots after scanning the microarray using a wavelength of 633 nm, while positive spots at 543 nm (Cadaverine) confirmed that spotting had occurred.

In order to address the microarray quality issue, we introduced three additional features, including array design, reference marker, and printing marker. To match the multi-well incubation gasket, we printed up to 14 identical subarrays per slide, as illustrated in Fig 3A. Each subarray was further divided into three identical segments, separated by printed rows of b-BSA. The use of b-BSA as reference marker (positive control) was critical for i) evaluating technical quality control (intra- and inter-array variations), ii) detecting technical outlier arrays due to local surface defects, and iii) defining the beginning and the end of each individual array segments and individual subarrays. The mean CV for b-BSA was found to be 7%, based on in total 1500 spots (corresponding to 3 slides/day for 2 days, 10 subarrays/slide, 25 replicate spots/subarray). The fact that each subarray was designed into three identical segments meant that one or even two segments could be deleted due to e.g. local surface defects, and data could still be obtained (data not shown).

The microarray spotter has no built-in mean to control that each individual spot has been successfully dispensed during the printing process. In order to bypass this limitation, we added low concentrated Alexa Fluor 555-Cadaverine to the printing solution as a positive printing marker. Thus, the observed lack of signal intensity at 633 nm for a given antibody could be explained by either that no protein antigen was present in the sample and/or that the antibody was not printed due to technical issues (Fig 3B). However, by adding Cadaverine, a signal or no signal at 543 nm helped us resolve this key issue (Fig 3B). This will further improve the microarray quality control.

### Sample quality control

We evaluated the reproducibility of our sample handling step, focusing on the biotinylation of crude serum samples. To this end, the same batch of pooled human serum was biotinylated and profiled on a 121-plexed antibody array layout (layout B) while varying i) the batch of biotin, ii) the user, and/or iii) day of analysis (Table 2). The reproducibility was evaluated in terms of CV of the observed signal intensities for the captured proteins, thus running the entire microarray assay. The mean CV for inter-run and inter-batch was found to be 19.7% and 16.3% (see Table 2 for assay set-up definition). It should be noted that raw, un-normalized data was used. Thus, the CV values also harbored array-to-array variability (estimated to be in the range of 11 to 13%, see below). Hence, the reproducibility of the labeling step of crude serum samples was found to be high.

**Table 2:** Sample labeling QC.

Mean CV (%)	Raw data
Inter Run*	19.7
Inter Batch**	16.3

* Calculated for eight separate labelling rounds performed by two users on three different days. The samples were processed on two slides, generating 16 subarrays in total, and the mean CV value for all antibodies is calculated based on all arrays.

**Calculated for four separate labelling rounds, two rounds for each biotin batch, performed by two users. The samples were processed on two slides, generating eight arrays in total, and the mean CV value for all antibodies is calculated based on all arrays.

### Microarray assay quality control

The performance of the microarray assay was improved by further optimizing i) the choice of blocking buffer (1% (v/v) Tween-20 and 1% (w/v) fat-free milk in PBS vs. 5% (v/v) fat-free milk in PBS) (reducing non-specific background binding), and ii) the sample incubation time (2h vs. 1h) (increasing signal intensities and signal to noise ratios) (see Material and Methods). In addition, three types of QC samples, denoted QC_ref_, QC_label_ and QC_norm_, were introduced in order to further standardize the set-up (see Material and Methods).

**Fig 4:**
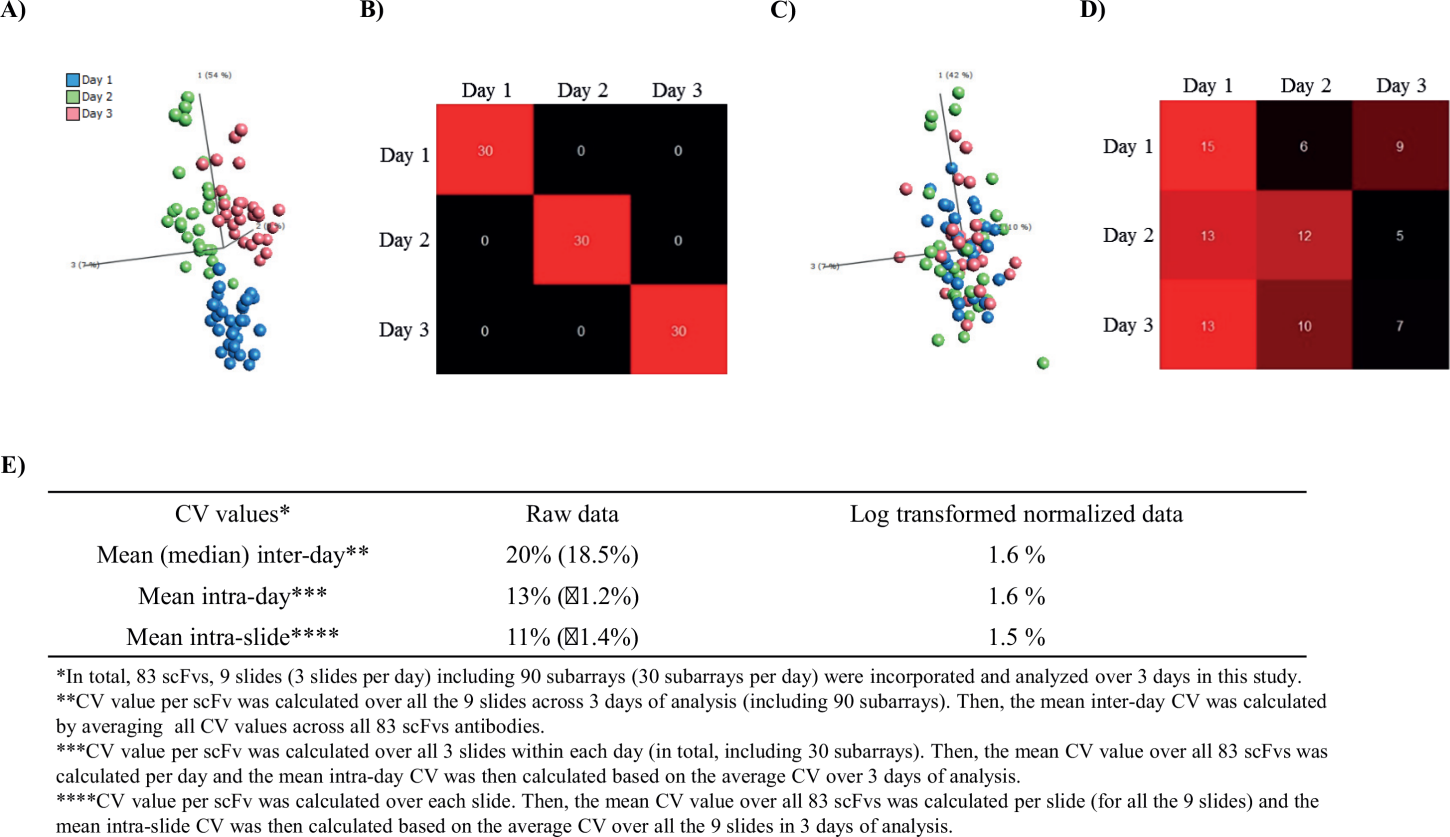
Day-to-day variation before and after normalization. (**A**) Unsupervised PCA analysis of un-normalized log2 transformed raw data. The samples were colored with respect to different rounds (days) of analysis. (**B**) Random Forest supervised classification differentiates between different days of analysis. (**C**) Unsupervised PCA analysis of processed data. The data was normalized using the “subtract by group mean” approach. (**D**) Random Forest classifier applied after normalization step. (**E**) Systemic technical variation, expressed in terms of CV, with respect to mean (median) inter- and intra-day as well as mean intra-slide variations.

With these new settings in place, we evaluated the source and effect of systemic technical variations on our microarray data. To this end, a 94-plex antibody microarray (83 spotted antibodies passed QC) was used (layout A) to profile the same pooled human serum sample on 90 subarrays, processed on 9 slides, and analyzed over 3 days (3 slides/day) (Fig 4). Principle component analysis (PCA) of un-normalized log2 transformed data revealed three distinct groups of samples associated with day of analysis (Fig 4A). Furthermore, adopting a random forest classifier, also demonstrated a distinct dependence of the day of analysis (Fig 4B), with an out-of-bag (OOB) estimate of error rate of 0%. Hence, the data showed that there was a systematic technical variation associated with different days of analysis.

In an attempt to reduce this variation, we employed our current standard approach to neutralize day-to-day variations, namely “subtract by group mean”. PCA analysis of the normalized data, showed that the day-to-day dependence had been minimized (Fig 4C). In agreement, the RF classifier showed an OOB estimate error rate of 62%, further supporting the notion that the normalization had minimized the day-to-day variations (Fig 4D). In addition, one-way analysis of variance (ANOVA) analysis confirmed that no statistically significant (q < 0.05) proteins correlated with the days of analysis. The overall mean CV of the observed signal intensities (captured proteins) was found to be 20% for raw, un-normalized data, but only 1.6% after normalization (log2 transformed data) over all days of analysis (Fig 4E). Further, the mean intra-day and intra-slide CV was found to be 13 and 11% for raw, un-normalized data, but only 1.6 and 1.5% after normalization. Taken together, the data showed that the day-to-day variations could be handled by data normalization.

### Biostatistics–data pre-processing

**Fig 5:**
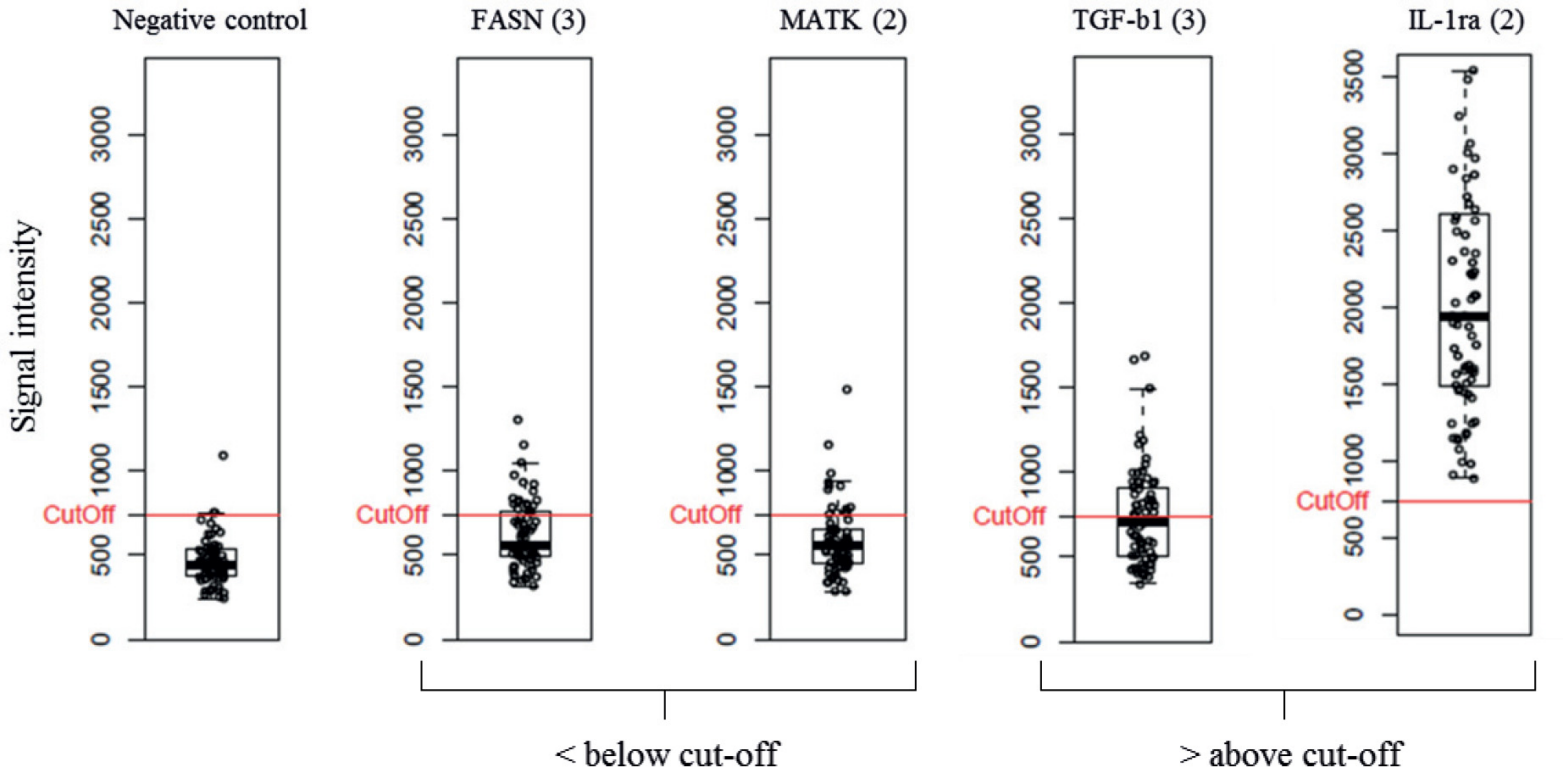
Limit of detection. Boxplots for 5 antibody intensities over all the analyzed samples. Each data point represents one sample. A cut-off limit was established based on the mean negative control signal (PBS) across all the samples plus 2 standard deviations. Each analyte, from which the mean signal intensities were found to be below the LOD in > 70% of samples was removed from the data (e.g. FASN (3) and MAKT (2)). The red line corresponds to the cut-off limit.

We standardized and/or further evaluated the data pre-processing procedure. Prior to the key step, array data normalization, the data was subjected to three actions. First, any background signals were subtracted from the measured signals in order to remove any non-biological contributions. Second, the mean value of the replicate spots for each antibody was calculated, after removing any outlier spots. Third, any antibodies below limit of detection, defined as mean blank signal across all samples plus 2 standard deviations in >70% of the samples, were identified and excluded from the data set, as illustrated in Fig 5.

Next, the data normalization step was evaluated. To this end, a 195-plex antibody microarray was used (layout C) to profile 224 serum samples (151 diseased and 57 controls) and 16 QC_ref_ serum. In total, 16 slides, with 14 subarrays each, were run during 2 days (8 slides/day). In total, nine different normalization strategies were tested and compared to i) our currently adopted normalization method, “subtract by group mean” strategy combined with “semi-global normalization” as well as ii) un-normalized log2 transformed raw data (Table 1).

First, we compared the effect of Quantile, VSN (local/global), and LOESS (local/global) normalization on the data set to raw un-normalized log2 transformed data by the means of intensity distributions in density plots, boxplots, and meanSdPlots (S1–S3 Figs). The density- and box-plot data showed that the array-to-array variations decreased in the order of Quantile < LOESS < VSN < un-normalized log2 (S1 and S2 Figs). Further, both local VSN and local LOESS normalization methods resulted in two more clearly separated sample distributions associated with different days of analysis, indicating that these methods (when applied locally) were unable to correct for day-to-day variation. In contrast, global VSN and global LOESS normalized data indicated that the variation across samples were more homogeneously distributed, outlining a better normalization (S1 and S2 Figs). In the case of Quantile, the results showed that the data was strongly transformed, illustrated by the fact that the normalized data displayed the same total intensity across all the arrays (S1 and S2 Figs). The meanSdPlots showed that the normalization methods reduced the variance-mean dependency from ~0.8 in un-normalized log2 transformed data to just above 0.5 (local LOESS and local VSN), just below 0.5 (global LOESS and global VSN), or from 0.4 to 0.6 (Quantile) (S3 Fig). In particular global LOESS showed the most consistent mean-variance dependency. Hence, the data suggested that global LOESS and global VSN resulted in the best normalization, but that some additional array-to-array normalization would be required.

**Fig 6:**
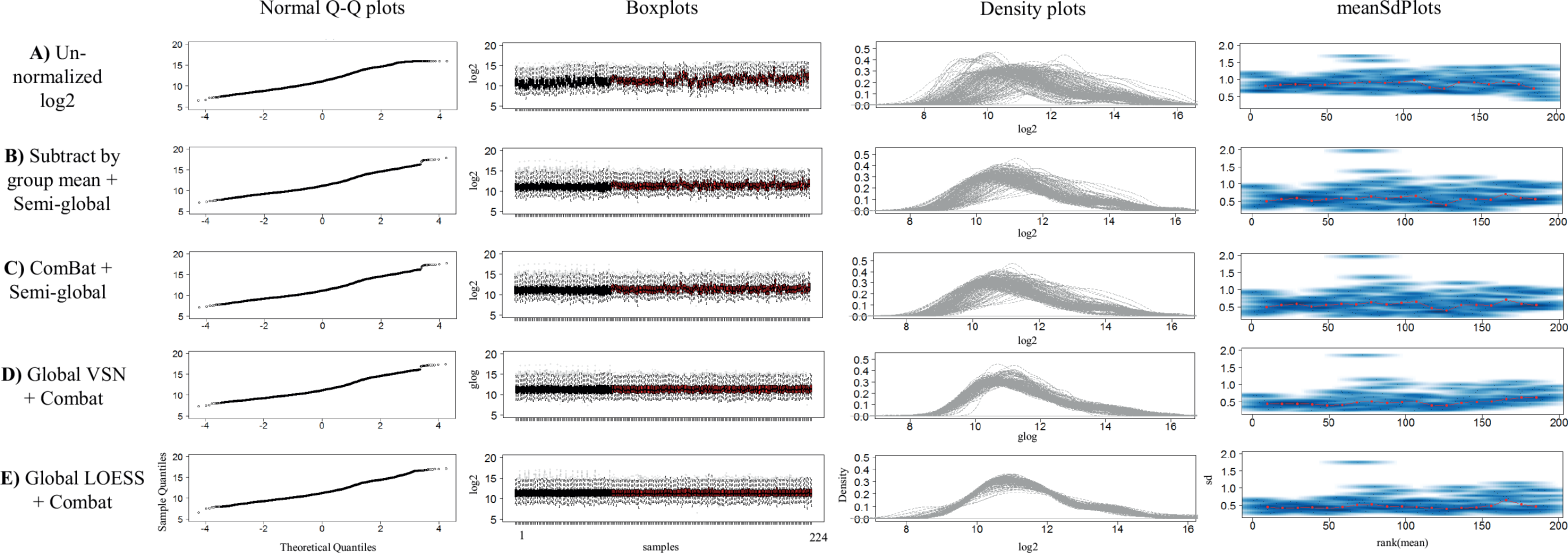
Evaluation of different normalization procedures. Normal Q-Q plots, boxplots, density plots and meanSdPlots were used as qualitative measures in order to compare the log intensity distribution of samples output after normalization. **(A)** Un-normalized log2 data. **(B)** Subtract by group mean + semi-global normalization. **(C)** ComBat + semi-global normalization. **(D)** Global VSN + Combat normalization. **(E)** Global LOESS + Combat normalization.

In order to address this additional normalization need, we proceeded and tested three additional methods, including (i) global VSN Combat normalization, (ii) global LOESS ComBat normalization, and (iii) ComBat semi-global normalization, and compared the output with that of our current adopted normalization method, i.e. subtract by group mean combined with semi-global normalization. Again, un-normalized log2 transformed raw data was used as reference. To start with, the methods were evaluated by the means of intensity distributions in Q-Q plots, density plots, boxplots, and meanSdPlots (Fig 6). When comparing the intensity distribution/variation of the samples, the results clearly showed that the array-to-array variation was reduced by all normalization methods as compared to un-normalized data (Fig 6). The normal Q-Q plots and density plots showed that global LOESS ComBat normalization resulted in the most symmetric and bell-shaped distributions, i.e. closest to normal distribution. Similarly, the meanSdPlots indicated that global LOESS ComBat displayed the most consistent mean-variance dependency (straightest line).

**Fig 7:**
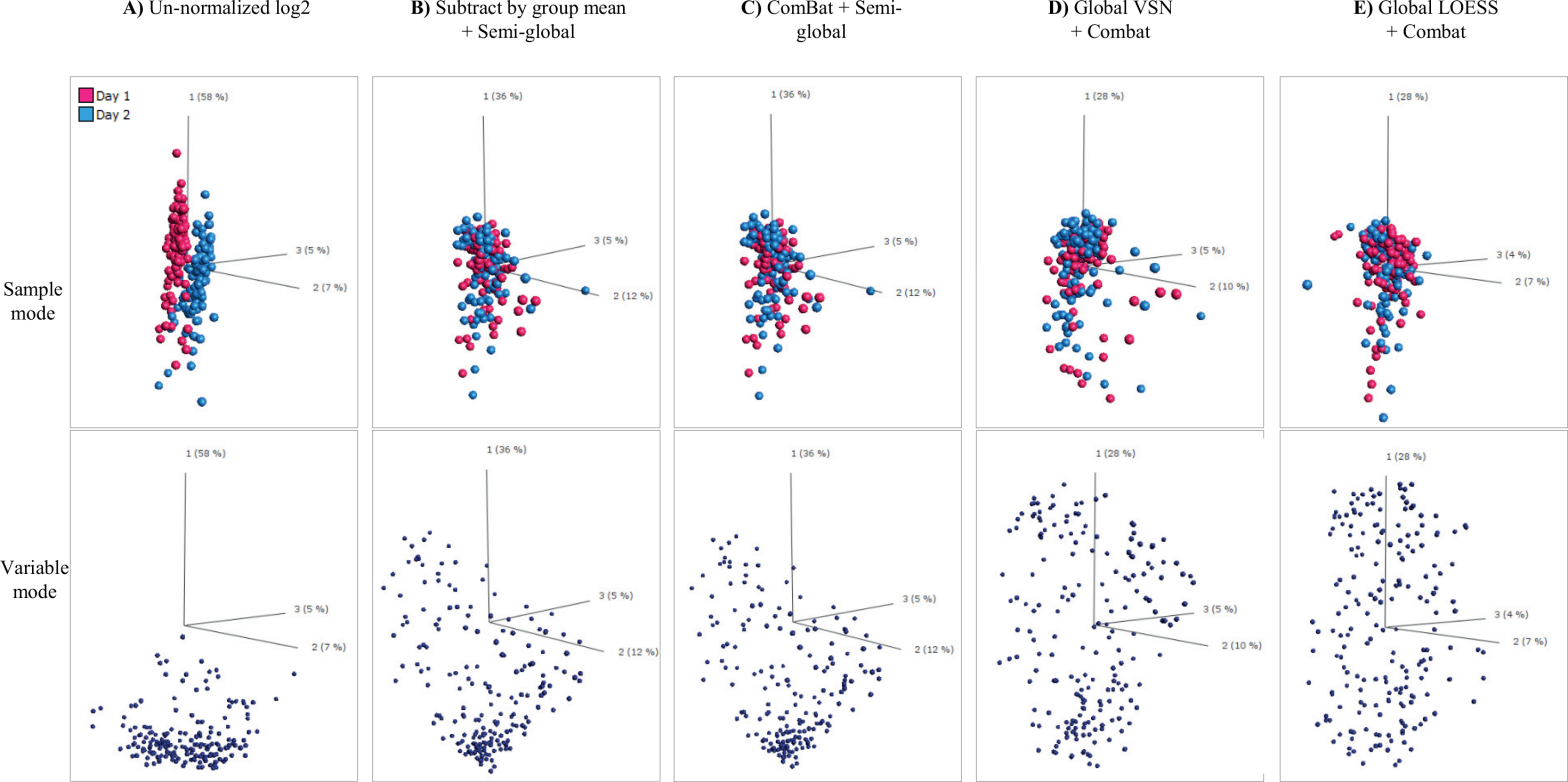
Evaluation of the effect of different normalization approaches on samples and variables in the data, shown in both sample mode and variable mode. **(A)** Un-normalized log2 data. **(B)** Subtract by group mean + semi-global normalization. **(C)** ComBat + semi-global normalization. **(D)** Global VSN + Combat normalization. **(E)** Global LOESS + Combat normalization

Next, we evaluated the normalization approaches with respect to sample PCA plots (Fig 7). A clear dependence of day of analysis (two) was observed for un-normalized data (Fig 7A, sample mode). The day-to-day dependence was noticeably reduced by all four normalization methods (Fig 7B–7E, sample mode).

Along the same line, we then assessed the relative impact of the normalization approaches on variables (scFv) using variable PCA plots (Fig 7, variable mode). A majority of variables displayed similar behavior in un-normalized data (Fig 7A). This characteristic behavior of variables was best retained using either ComBat semi-global (Fig 7C) or our currently adopted normalization method (Fig 7B). However, the behavior was dramatically changed using the other normalization methods (Fig 7D and 7E), and in particular global LOESS ComBat (Fig 7E).

To further examine the effect of the normalization on day-to-day variation, we performed a two-group comparison (day 1 vs. day 2), based on either i) different samples (i.e. all samples but QC_ref_) or ii) identical samples (i.e. technical replicates of the QC_ref_ samples), using t-test (Table 3). When using different samples, the data showed that all normalization methods appeared to eliminate the detectable day-to-day variations (Table 3). However, removing the sample dependency and using only technical replicates of the sample (QC_ref_), showed a refined view. The number of statistically significant (q < 0.01) scFv decreased in the order of global LOESS Combat (n = 26) < Global VSN Combat < ComBat semi-global < Subtract by group mean plus semi-global < un-normalized (n = 158) (Table 3). Hence, all normalization methods were found to significantly reduce the day-to-day variation, but in a method dependent manner.

**Table 3:** Evaluation of different normalization methods. To this end, microarray data was used to assess the No of statistically significant scFvs (n_total_ = 195) when comparing day 1 vs. day 2, using a cut-off value of either q <0.05 or q <0.01 as well as q < 0.05 plus FC > 1.5.

Normalization method	All subjects except QC-ref samples, at q < 0.05	Only QC-ref samples, at q < 0.05	Only QC-ref samples, at q < 0.01	Only QC-ref samples, at q < 0.05 plus FC > 1.5
Un-normalized log2 transformed data	163	167	158	43
Subtract by group mean + semi-global	0	61	37	6
ComBat + semi-global	0	59	35	5
Global VSN + Combat	0	46	29	4
Global LOESS + Combat	0	47	26	6

Next, we investigated the impact of the normalization methods on an application, by comparing the number of deregulated proteins when comparing healthy vs. diseased samples, in terms of fold change (FC). A FC > 1 or FC > 1.1, with and without a cut-off value of q <0.05 was applied (Table 4). The results showed that the number of down- vs. up-regulated proteins (as expressed in terms of deregulated scFv antibodies) was much smaller in ComBat semi-global and Subtract by group-mean plus semi-global, while the regulation was symmetrically distributed in global VSN Combat and LOESS ComBat normalized data, irrespective of the chosen cut-off. A similar trend was observed when comparing two subgroups within the diseased samples only (S2 Table). To examine this feature in more detail, we determined the frequency when all scFv antibodies against the same protein gave similar results with respect to up- vs. down regulation (FC > 1), denoted number of complete matches per target protein (Table 4). The number of complete matches per protein (n_total_ = 47) was higher in both ComBat semi-global and Subtract by group mean plus semi-global than in global VSN Combat and LOESS ComBat normalized data sets. Again, the same behavior was observed when comparing two subgroups with the diseased samples only (S2 Table). The data thus indicated on significantly different effects of the normalization methods on the data, outlining a more preferred behavior of ComBat semi-global and Subtract by group mean plus semi-global normalization.

**Table 4:** Evaluation of different normalization processes. To this end, microarray data for diseased vs. healthy samples was used and compared with respect to No. of down-regulated scFvs antibodies and No. of complete matches per target molecule, using a fold change (FC) filter of either FC > 1 or FC > 1.1 and with and without a cut-off value of q <0.05.

Diseased vs. Healthy	Subtract by group mean + semi-global norm	ComBat norm + semi-global norm	Global VSN + Combat norm	Global LOESS + Combat norm
No. of down-regulated scFvs at FC > 1	38 of 195	37 of 195	91 of 195	99 of 195
No. of down-regulated scFvs at FC > 1 and q <0.05	21 of 151	20 of 150	58 of 135	61 of 119
No. of down-regulated scFvs at FC > 1.1 and q <0.05	21 of 146	20 of 145	53 of 125	57 of 108
No. of complete matches* per target molecule (n_total_ = 47)	32	33	23	21

* Each protein analyte was targeted by 2–9 individual scFv clones. A complete match is defined as when all scFvs per target gave similar results with respect to up- vs. down regulations, using a fold change (FC) filter of FC > 1.

**Fig 8:**
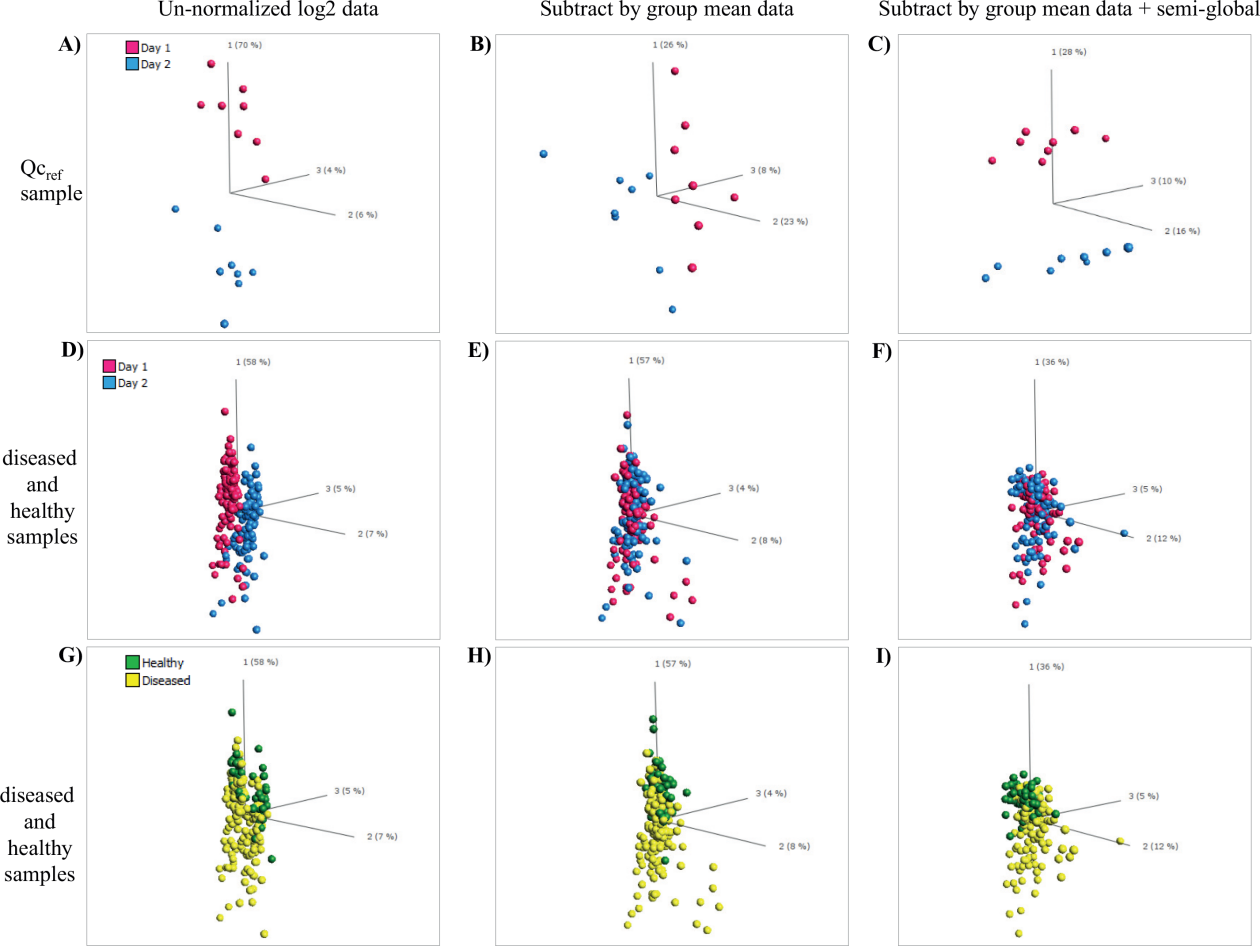
Effect of the normalization on sample variability. Un-normalized log2 data was compared with subtract by group mean normalized data as well as with subtract by group mean + Semi-global normalized data. The sample cohort included QC_ref_ samples as well as diseased and healthy samples. (A to C) QC_ref_ samples analyzed on two days. (D to F) Diseased and healthy samples analyzed on different days (mapped for day 1 vs. day 2). (G to I) Diseased vs. healthy samples analyzed on different days (mapped for diseased/healthy).

Finally, focusing on the two best normalization methods, Subtract by group mean plus semi-global normalization and ComBat semi-global, we used PCA to investigate the variability between all samples (healthy, diseased, and QC_ref_). Using Subtract by group mean plus semi-global normalization as showcase, the data clearly showed that the day-to-day variation had been reduced, as seen for both QC_ref_ day 1 vs. day 2 (Fig 8A–8C), and for diseased and healthy samples day 1 vs. day 2 (Fig 8D–8F). In the latter plot, this became much clearer when the samples were mapped as healthy and diseased (Fig 8G–8I). Notably, similar results were observed for Combat semi-global normalized data (S4 Fig). It should also be noted that the core biological differences, with respect to the top 50 differentially expressed proteins (represented by antibody clones), between diseased and healthy samples were maintained after normalization compared to un-normalized data (S3 Table). The data showed that 32 of 50 clones were retained, although their q-values dropped (from 10^−9^–10^−14^ to 10^−7^–10^−13^) and their individual order changed. Furthermore, 18 of 50 clones were replaced, but these new clones were included irrespective of the two normalization methods adopted. Taken together, our data showed that ComBat semi-global and Subtract by group mean plus semi-global normalization were the two preferred normalization methods.

### Biostatistics–condensed biomarker panels

Defining a condensed biomarker signature providing the best classification of two groups is essential in array based discovery applications. To this end, we for the first time compared and evaluated four different approaches for biomarker panel condensation, including i) selection based on Wilcoxon p-values, ii) backward elimination using SVM (BE-SVM), iii) modified backward elimination using SVM (consensus approach) (SVMc), and iv) RF. Two independent samples cohorts, cohort 2 and 3, randomly divided into a training set and a test set, were used as sample sets. The samples were analyzed on 351-plex antibody arrays (layout D).

First, condensed biomarker signatures of a fixed length (N = 25) of the top-ranked biomarkers were defined using each of the methods (Table 5). The classification performance of the signatures was then tested and described in terms of ROC AUC values. The signatures were defined based on the training set, and evaluated on the separate test set, using a linear SVM or RF. This entire procedure was repeated two times using a 3-fold cross validation scheme to obtain more reliable test performances (S5 Fig). The results showed that condensed signatures based on p-value ranking displayed the lowest AUC values, indicating that this approach was less effective in capturing collective effects (i.e. orthogonal information) among the biomarkers, in particular for cohort 2. In contrast, the BE-SVM and SVMc approaches generated the highest AUC values. Further, these two approaches generated similar results on both sample cohorts, indicating that the potential overtraining associated with SVM was not significant in the evaluated data sets. The RF method was able to find a high performing signature for cohort 2, but not for cohort 3. When comparing which biomarkers were included in the condensed signatures, BE-SVM and SVMc generated very similar lists, with an overlap of 15 or 17 biomarkers (Table 5). In contrast, the p-value ranking and SVM (both approaches) generated very different lists, with only 3 or 4 biomarkers overlap (Table 5). Thus, the data again indicated that the collaborative effects might be less well captured by the p-value ranking method. In this context, it might be of interest to note that there was in average 6 biomarkers in the SVM signatures that had Wilcoxon p-values > 0.1 (uncorrected p-values) (data not shown). The signature overlap was, however, more similar between the p-value ranking and RF with 15 and 20 markers for cohort 2 and 3, respectively. Hence, the choice of method will be important for defining the nature of the best performing classifier, i.e. the condensed biomarker signature.

**Table 5:** Evaluation of four feature selection methods for defining a condensed biomarker signature for classification of two groups, using a 3-fold cross validation scheme repeated twice. The length of the biomarker signature was set to 25. A linear SVM classifier was used to assess the performance in all cases, except for RF (RF), where random forest was used both for the ranking and the final AUC calculation. AUC values are given together with standard deviations. The biomarker overlap was defined as the number of common biomarkers between two signatures.

Method	Sample cohort 2				Sample cohort 3			
		Biomarker overlap				Biomarker overlap		
		BE-SVM	SVMc	RF		BE-SVM	SVMc	RF
	ROC AUC				ROC AUC			
p-value	90.1 (2.3)	3	4	15	90.6 (1.3)	8	7	20
BE-SVM	95.9 (3.2)		17	6	93.5 (1.1)		15	9
SVMc	96.7 (2.1)			7	92.1 (0.5)			9
RF (SVM)	94.5 (1.3)				91.7 (0.8)			
RF (RF)	95.1 (2.1)				90.9 (0.8)			

Second, we re-ran the condensation methods, and defined the best performing condensed biomarker signatures, but without fixing the signature length (Table 6). The signatures were defined and tested on sample cohort 3 (the largest cohort). The sample cohort was split into training (1/3 of all samples, to define the biomarker ranking list), validation (1/3, to define the optimal signature length), and test (1/3, to test the defined signature) sets. The data showed that the signatures varied in length, from 30 (p-value and SVM) to 45 (RF). Again, the results showed that the p-value method resulted in the lowest ROC AUC value, and the two SVM methods presented the best AUC values. In general, the ROC AUC values were retained or improved going from a fixed biomarker signature length to the most optimal length, in particular for RF. Hence, the choice of method will be essential for defining the nature and length of the best performing classifier, i.e. the condensed biomarker signature.

**Table 6:** Evaluation of four methods for defining a condensed biomarker signature for classification of two groups. Once the biomarker signature of the optimal length was defined using the validation set, the classifier was re-trained, using both the training and the validation set and finally tested on the test set. Sample cohort 3 was used.

Method	Biomarker length	ROC AUC	
		Validation set	Test set
p-value	30	92.2	90.6
BE-SVM	30	93.6	92.7
SVMc	40	93.9	94.0
RF (SVM)	45	93.9	93.1

## Discussion

The number of high-performing antibody microarray set-ups at hand is still very low, which is explained by the fact that systematic cross-disciplinary efforts, addressing all of the key methodological areas involved in an array set-up (Table 1), must be pursued in such a developmental work [2, 8–10]. Here, we have presented the next generation of our recombinant antibody microarray technology platform for clinical immunoproteomics. In this work, we have further advanced our previous array platform version [4, 7, 22] by continuing our interdisciplinary work and addressing a set of points, ranging from small technical refinements (e.g. re-optimized blocking solution and sample incubation time) to key essential process improvements (e.g. array data normalization and biomarker panel condensation) (Table 1). To the best of our knowledge, this indeed represents one of the first studies in which pre-processing of antibody microarray data (e.g. normalization) and condensation of biomarker panels have been studied in great detail.

Among the technical array attributes, the specificity of the dispensed antibodies is a vital feature [14]. The specificity of the antibodies used *per se* have been extensively addressed in recent work by us [4, 7, 11, 16–21] (Säll *et al*, manuscript in prep.). But to incorporate a built-in specificity control step in the microarray assay, we have included several antibody clones directed against the same protein antigen, but targeting different epitopes. This is essential, as some antibodies might lose their reactivity caused by i) epitope masking, e.g. caused by the biotinylation of the sample [7], and complex formation, and/or ii) (partial) denaturation when arrayed onto the solid support. Albeit our arrayed antibodies displayed high on-chip activity, and that matching antibody clones displayed similar and highly reproducible reactivity patterns, i) the observed fold changes differed somewhat between matching antibody clones (Fig 2C), and ii) some antibodies were found to be less reactive, at least when some samples were profiled (e.g. Table 4). Thus, without such a built-in feature, the risk for including false-negative, and potentially false-positive signals is evident. In future work, the entire antibody production process and subsequent quality control steps, such as mono-dispersity, will also be addressed, which could improve the antibody reagent source and thereby the antibody array platform even further.

Reproducibility is another central array attribute, which has been a main issue for many antibody array-based set-ups [8]. Here, the reproducibility of the entire assay set-up including microarray printing, microarray assay, and data pre-processing, expressed in terms of the mean intra-day and intra-slide CV, was found to be 13 and 11% respectively for raw, un-normalized data, but only 1.6 and 1.5% respectively after normalization. These values are highly competitive, and required when aiming for clinical immunoproteomics targeting truly low-abundant proteins (pg/ml range) [2, 8, 9].

The interplay between arrayed antibodies and the surface, modulated by the spotting buffer [34], is essential for antibody functionality and spot features [7, 22, 35, 36]. Frequently, excess of antibodies have been dispensed in order to make sure that the spots are fully saturated. Our data implied that the spots grew from the center and outwards with increasing antibody concentration, and that the antibodies appeared to populate the spots in a clone dependent manner. The spots stopped growing at a diameter of about 130 μm, even if the antibody concentration was increased further, a feature that, at least in part, might be explained by the properties of the solid support and/or spotting buffer. This means that adding even more surplus of antibodies only resulted in loose multilayers of antibodies that were washed off in the subsequent washing step, causing adverse smearing and tailing effects. Consequently, the data demonstrated that it was critical to optimize the spotting concentration for each antibody to promote fully saturated spots with distinct, optimal features, directly impacting the subsequent spot detection and quantification steps. Future work will be required to explore the importance of the sample format, i.e. serum vs. urine vs. tissue extracts (and precise analyte concentration), on the amount of spotted antibody.

A major effort was placed on addressing the data handling step, and in particular normalization of the array data and identification of condensed biomarker panels. In fact, this is one of the very first studies addressing these key steps in great detail. Although the choice of normalization method is essential [37, 38], antibody (protein) microarray data normalization have, so far, received very little attention, as judged by the number of published papers on the subject, see e.g. [4, 39–41]. In fact, no standard protocol for protein microarray data handling at large, including both pre-processing and subsequent data analysis, have yet been established [9]. Compared to large gene expression arrays, antibody microarrays are focused, containing more relevant targets of which many could be expected to be deregulated [8, 42]. From an analytical point of view, this difference in number of targets reduces the risk for false positive and false negative findings as well as the extent of multiple testing correction needed, but makes the key normalization step much more challenging. As for example, most normalization approaches, such as LOESS and Quantile rely on the assumption that i) a majority of the analytes are not differentially expressed, and ii) that there is a symmetry in the expression levels of the up- and down-regulated analytes [43–45]. However, these two assumptions cannot readily be made for focused antibody microarrays, such as ours [8, 9]. In agreement, our evaluation of normalization methods also showed that the impact of the LOESS and Quantile approaches on the data was too strong (unfavorable), making them a less attractive choice. Although the aim is to eliminate systematic technical variations, such as day-to-day variations and array-to-array variations, it is essential not to transform the data too much so that also subtle biological changes are eliminated [38, 43].

In the data sets tested here, we identified both day-to-day and array-to-array variations that should be handled via the normalization step. While all the tested normalization methods were found to reduce at least some of the variations, the manner in which and to what extent this was accomplished, differed considerably. Of the normalization methods tested, we found two approaches, subtract by group mean plus semi-global (our currently adopted approach) and ComBat semi-global, to perform the best. This means that these normalization procedures were capable of handling both day-to-day and array-to-array variations, but with the smallest transformation of the data set and (potentially) maintaining biological differences at large in the data set. In future work, any of these methods could be applied, and the precise choice would have to be determined data-set by data-set.

Deciphering condensed biomarker panels, going from several hundreds to about twenty markers or less, providing the best discriminatory power for the question at hand, e.g. diagnosis, will be essential in the development of novel tests [8]. In the end, a condensed panel, composed of a small number of biomarkers, with each biomarker providing unique, orthogonal information, is desired.

Finding a condensed panel of biomarkers that performs optimally for a given diagnostic problem can be viewed as a feature selection task in machine learning. Here, the diagnostic problem is transformed into a classification problem using all available biomarkers as features. The task is to find a reduced set of features that results in optimal (or near-optimal) classification performance. Here we used the ROC AUC value as a performance measure. The p-value ranking was found to generate biomarker panels displaying the worst AUC values, which could be explained that the markers were selected based on p-values and not whether they provided orthogonal information. This means that many of the selected markers might have provided similar information. Biomarkers selected based on p-values are therefore likely better to reflect the disease and disease state rather than reflecting the best classifier.

Further, care has to be taken to avoid overtraining, here meaning the problem of determining a condensed biomarker panel too specialized on one cohort, thereby lacking the necessary generalization to other cohorts of the same diagnostic problem. The overtraining problem is typically present in situations with small sample sizes and a large number of features (biomarkers). The classification methods used in the feature selection process are selected to have as few tunable parameters as possible, to avoid overtraining on method parameters. In this study, we did not see any significant signs of overtraining which might be due to e.g. large sample sizes, but in future data sets this might become essential. It should be noted that any further refinement of a condensed signature should be validated using a novel independent sample cohort(s). Taken together, the results showed that we have defined two excellent ways of defining condensed biomarker signatures, namely SVM and SVMc, the latter method having a built-in function to avoid overtraining. Depending on the nature of the data set, RF might also be a viable option, while the p-value ranking methods is less recommended.

Despite the current advances, some features could be subjected to even further optimizations. As for example, the range of specificities included on the array is critical for defining the resolution at which each sample can be profiled. Here, we used up to 351-plex arrays, but have in recent applications used 395-plex antibody arrays [46], and we have up to 900-plex antibody arrays in the pipeline (Wingren *et al*, unpublished observations). Other issues to resolve could include, but are not limited to, orientated antibody immobilization for improved functionality [47], assay automation, next generation of user friendly software for big data analysis, standardized repositories for protein microarray data, and absolute quantification.

Taken together, we have continued our interdisciplinary efforts, and presented the next generation of our recombinant antibody microarray technology platform for clinical immunoproteomics. This platform could pave the way for the next wave of clinical applications, with great potential for biomarker discovery and clinical endeavors, such as diagnosis, prognosis, and classification.

## Supporting Information

S1 Fig Evaluation of different normalization procedures. Density plots were used as qualitative measures in order to compare the log intensity distribution of samples output with respect to log2 transformed data, Local LOESS, Local VSN, Global LOESS, Global VSN and Quantile normalizations.
(EPS)

S2 Fig Evaluation of different normalization procedures. Boxplots were used as qualitative measures in order to compare the log intensity distribution of samples output with respect to log2 transformed data, Local LOESS, Local VSN, Global LOESS, Global VSN and Quantile normalizations.
(EPS)

S3 Fig Evaluation of different normalization procedures. MeanSdPlots were used as qualitative measures in order to compare the log intensity distribution of samples output with respect to log2 transformed data, Local LOESS, Local VSN, Global LOESS, Global VSN and Quantile normalizations.
(EPS)

S4 Fig Effect of the Combat plus semi-global normalization on the sample variability. The sample cohort included QC_ref_ samples as well as diseased and healthy samples. i) QC_ref_ samples analyzed on two days. ii) Diseased vs. healthy samples analyzed on different days (mapped for day). iii) Diseased vs. healthy samples analyzed on different days (mapped for diseased/healthy).
(EPS)

S5 Fig Schematic outline of how methods for defining a condensed biomarker signature were evaluated. The data set, composed of two groups–healthy (H) and disease (D)—was randomly divided in two three subsets. Condensed biomarker signatures of a fixed length (N = 25) of the top-ranked biomarkers were defined using each of the feature selection methods (p-value, BE-SVM, SVMc, and RF). The classification performance of the signatures was then tested and described in terms of ROC AUC values. The signatures were defined based on the training set, and evaluated on the independent test set, using a linear SVM or RF. This entire procedure was repeated two times using a 3-fold cross validation scheme to obtain more reliable test performances
(EPS)

S1 Table Antigens targeted on the antibody microarray. The specificity, affinity (normally in the nM range), and on-chip functionality of all of these phage display derived scFv antibodies were ensured by using i) stringent phage-display selection and screening protocols (using different sample formats, ranging from pure proteins and mixtures of pure proteins to crude samples) (16), ii) multiple clones (1 to 9) per protein, and iii) a molecular design, adapted for microarray applications (14). In addition, the specificity of several selected antibodies (marked with an *) have been further validated using pure proteins, mixtures of pure proteins, as well as well-characterized, standardized serum samples (with known levels of the targeted analytes, spiked with known level of specific protein(s) and/or specific protein(s) depleted), and/or orthogonal methods, such as mass spectrometry (affinity pull-down experiments), ELISA, MesoScaleDiscovery assay, and cytometric bead assay, as well as using blocking experiments [4, 7, 11, 17–21].
(DOCX)

S2 Table Evaluation of different normalization processes. To this end, microarray data for diseased group 3 vs. group 1 samples was used and compared with respect to No. of down-regulated scFvs antibodies and No. of complete matches per target molecule, using a fold change (FC) filter of FC > 1.
(DOCX)

S3 Table Top 50 differentially expressed proteins (represented by their matching antibody clone) for diseased vs healthy controls, before and after normalization. (DOCX)

## Abbreviations

CV: coefficient of variation
scFv: single-chain Fragment variable
PCA: principal component analysis
QC: quality control
RF: random forest
SOP: standard operating procedures
SVM: support vector machine
