# Supplementary material for: Technical Advances of the Recombinant Antibody Microarray Technology Platform for Clinical Immunoproteomics
Source: PLoS One. 2016 Jul 14;11(7):e0159138. doi: 10.1371/journal.pone.0159138 (PMC4944972; doi:10.1371/journal.pone.0159138)
Supplement: S1 Table — The specificity, affinity (normally in the nM range), and on-chip functionality of all of these phage display derived scFv antibodies were ensured by using i) stringent phage-display selection and screening protocols (using different sample formats, ranging from pure proteins and mixtures of pure proteins to crude samples) (16), ii) multiple clones (1 to 9) per protein, and iii) a molecular design, adapted for microarray applications (14). In addition, the specificity of several selected antibodies (marked with an *) have been further validated using pure proteins, mixtures of pure proteins, as well as well-characterized, standardized serum samples (with known levels of the targeted analytes, spiked with known level of specific protein(s) and/or specific protein(s) depleted), and/or orthogonal methods, such as mass spectrometry (affinity pull-down experiments), ELISA, MesoScaleDiscovery assay, and cytometric bead assay, as well as using blocking experiments [4, 7, 11, 17–21]. (DOCX) [file pone.0159138.s006.docx]

**S1 Table.** **Antigens targeted on the antibody microarray.** The specificity, affinity (normally in the nM range), and on-chip functionality of all of these phage display derived scFv antibodies were ensured by using i) stringent phage-display selection and screening protocols (using different sample formats, ranging from pure proteins and mixtures of pure proteins to crude samples) ([16](#_ENREF_16)), ii) multiple clones (1 to 9) per protein, and iii) a molecular design, adapted for microarray applications ([14](#_ENREF_14)). In addition, the specificity of several selected antibodies (marked with an *) have been further validated using pure proteins, mixtures of pure proteins, as well as well-characterized, standardized serum samples (with known levels of the targeted analytes, spiked with known level of specific protein(s) and/or specific protein(s) depleted), and/or orthogonal methods, such as mass spectrometry (affinity pull-down experiments), ELISA, MesoScaleDiscovery assay, and cytometric bead assay, as well as using blocking experiments [[4](#_ENREF_4), [7](#_ENREF_7), [11](#_ENREF_11), [17-21](#_ENREF_17)9.

| Protein | Full name | No of antibody clones |
| --- | --- | --- |
| AGAP-2 | Arf-GAP with GTPase, ANK repeat and PH domain-containing protein 2 | 2 |
| AIP-3 | Membrane-associated guanylate kinase, WW and PDZ domain-containing protein 1 | 2 |
| Angiomotin | Angiomotin | 2 |
| APLF | Aprataxin and PNK-like factor | 2 |
| Apo-A1 | Apolipoprotein A1 | 3 |
| Apo-A4 | Apolipoprotein A4 | 3 |
| ARHGC-1 | Rho guanine nucleotide exchange factor 12 | 1 |
| ATP-5B | ATP synthase subunit beta, mitochondrial | 3 |
| BTK | Tyrosine-protein kinase BTK | 3 |
| C1 est. inh. | Plasma protease C1 inhibitor | 4 |
| C1q* | Complement C1q | 1 |
| C1s | Complement C1s | 1 |
| C3* | Complement C3 | 6 |
| C4* | Complement C4 | 4 |
| C5* | Complement C5 | 3 |
| CA19-9 | Carbohydrate antigen 19-9 | 2 |
| CaM kinase II subunit beta | Calcium/calmodulin-dependent protein kinase type II subunit beta | 2 |
| CaMK IV | Calcium/calmodulin-dependent protein kinase type IV | 2 |
| CaM-KK 1 | Calcium/calmodulin-dependent protein kinase kinase 1 | 2 |
| CD40 | CD40 protein | 4 |
| CD40L | CD40 ligand | 1 |
| CDK-2 | Cyclin-dependent kinase 2 | 2 |
| cGK 2 | cGMP-dependent protein kinase 2 | 2 |
| Chapsyn-110 | Disks large homolog 2 | 2 |
| CHP1 | Calcineurin B homologous protein 1 | 2 |
| CHX10 | Visual system homeobox 2 | 3 |
| CIMS | Peptide motifs | 19 |
| CKI-epsilon | Casein kinase I isoform epsilon | 2 |
| CT | Cholera toxin subunit B (Control) | 1 |
| Cystatin C | Cystatin-C | 4 |
| DCNL1 | DCN1-like protein 1 | 2 |
| Digoxin | Digoxin | 1 |
| DUSP7 | Dual specificity protein phosphatase 7 | 2 |
| DUSP9 | Dual specificity protein phosphatase 9 | 1 |
| EGFR | Epidermal growth factor receptor | 1 |
| Eotaxin | Eotaxin | 3 |
| Factor B* | Complement factor B | 4 |
| FAP-1 | Tyrosine-protein phosphatase non-receptor type 13 | 2 |
| FASN | FASN protein | 2 |
| FER | Tyrosine-protein kinase Fer | 2 |
| GAK | GAK protein | 2 |
| GEM | GTP-binding protein GEM | 2 |
| GLP-1 | Glucagon-like peptide-1 | 1 |
| GLP-1 R | Glucagon-like peptide 1 receptor | 1 |
| GM-CSF | Granulocyte-macrophage colony-stimulating factor | 4 |
| GNAI3 | Guanine nucleotide-binding protein G(k) subunit alpha | 2 |
| GOLPH6 | Golgi reassembly-stacking protein 2 | 2 |
| GPRK5 | G protein-coupled receptor kinase 5 | 1 |
| GRIP-2 | Glutamate receptor-interacting protein 2 | 3 |
| HADH2 | HADH2 protein | 2 |
| Her2/ErbB-2 | Receptor tyrosine-protein kinase erbB-2 | 2 |
| hINADL | InaD-like protein | 2 |
| HLA-DR/DP | HLA-DR/DP | 1 |
| hOTU1 | Ubiquitin thioesterase OTUB1 | 2 |
| HsHec1 | Kinetochore protein NDC80 homolog | 2 |
| HsMAD2 | Mitotic spindle assembly checkpoint protein MAD2A | 2 |
| hSpindly | Protein Spindly | 2 |
| Hucds1 | Serine/threonine-protein kinase Chk2 | 2 |
| IAP-2 | Baculoviral IAP repeat-containing protein 2 | 2 |
| ICAM-1 | Intercellular adhesion molecule 1 | 1 |
| IFN-γ | Interferon gamma | 3 |
| IgM | Immunoglobulin M | 5 |
| IL-10* | Interleukin-10 | 3 |
| IL-11 | Interleukin-11 | 3 |
| IL-12* | Interleukin-12 | 4 |
| IL-13* | Interleukin-13 | 3 |
| IL-16 | Interleukin-16 | 3 |
| IL-18 | Interleukin-18 | 3 |
| IL-1-ra | Interleukin-1 receptor antagonist protein | 3 |
| IL-1α* | Interleukin-1 alpha | 3 |
| IL-1β | Interleukin-1 beta | 3 |
| IL-2 | Interleukin-2 | 3 |
| IL-3 | Interleukin-3 | 3 |
| IL-4* | Interleukin-4 | 4 |
| IL-5* | Interleukin-5 | 3 |
| IL-6* | Interleukin-6 | 5 |
| IL-7 | Interleukin-7 | 2 |
| IL-8* | Interleukin-8 | 3 |
| IL-9 | Interleukin-9 | 3 |
| Integrin α-10 | Integrin alpha-10 | 1 |
| Integrin α-11 | Integrin alpha-11 | 1 |
| Itch | E3 ubiquitin-protein ligase Itchy homolog | 2 |
| JAK3 | Tyrosine-protein kinase JAK3 | 1 |
| Keratin19 | Keratin, type I cytoskeletal 19 | 2 |
| KRAS | GTPase KRas | 1 |
| KSYK | Tyrosine-protein kinase SYK | 2 |
| LDL | Apolipoprotein B-100 | 2 |
| Leptin | Leptin | 1 |
| Lewis x | Lewis x | 2 |
| Lewis y | Lewis y | 1 |
| Lin-7A | Protein lin-7 homolog A | 2 |
| Lumican | Lumican | 1 |
| MAPK1 | Mitogen-activated protein kinase 1 | 2 |
| MAPK8 | Mitogen-activated protein kinase 8 | 3 |
| MAPK9 | Mitogen-activated protein kinase 9 | 3 |
| MAPKK 2 | Dual specificity mitogen-activated protein kinase kinase 2 | 2 |
| MAPKK 6 | Dual specificity mitogen-activated protein kinase kinase 6 | 2 |
| MATK | Megakaryocyte-associated tyrosine-protein kinase | 2 |
| MCP-1* | C-C motif chemokine 2 | 9 |
| MCP-3 | C-C motif chemokine 7 | 3 |
| MCP-4 | C-C motif chemokine 13 | 3 |
| MUC-1 | Mucin-1 | 4 |
| Myomesin-2 | Myomesin-2 | 2 |
| N-NOS | Nitric oxide synthase, brain | 2 |
| ORP-3 | Oxysterol-binding protein-related protein 3 | 2 |
| Osteopontin | Osteopontin | 2 |
| OTU6B | OTU domain-containing protein 6B | 2 |
| OTUB2-1 | Ubiquitin thioesterase OTUB2 | 2 |
| P85A | Phosphatidylinositol 3-kinase regulatory subunit alpha | 2 |
| PAK-4 | Serine/threonine-protein kinase PAK 4 | 2 |
| PAK7 | Serine/threonine-protein kinase PAK 7 | 2 |
| Par-1c | Serine/threonine-protein kinase MARK1 | 2 |
| PAR-6B | Partitioning defective 6 homolog beta | 2 |
| PARP-1 | Poly [ADP-ribose] polymerase 1 | 1 |
| PGAM5 | Serine/threonine-protein phosphatase PGAM5, mitochondrial | 2 |
| PKB gamma | RAC-gamma serine/threonine-protein kinase | 2 |
| Pol Mu | DNA-directed DNA/RNA polymerase mu | 2 |
| PRD14 | PR domain zinc finger protein 14 | 3 |
| PRDM8-1 | PR domain zinc finger protein 8 | 2 |
| PRKCZ | Protein kinase C zeta type | 2 |
| Procathepsin W | Procathepsin W | 1 |
| Properdin* | Properdin | 1 |
| PSA | Prostate-specific antigen | 1 |
| PSD-95 | Disks large homolog 4 | 2 |
| PTK-6 | Protein-tyrosine kinase 6 | 1 |
| PTP-1B | Tyrosine-protein phosphatase non-receptor type 1 | 2 |
| RANTES | C-C motif chemokine 5 | 3 |
| RPS6KA2 | Ribosomal protein S6 kinase alpha-2 | 2 |
| R-PTP-delt | Receptor-type tyrosine-protein phosphatase delta | 2 |
| R-PTP-eta | Receptor-type tyrosine-protein phosphatase eta | 3 |
| R-PTP-kappa | Receptor-type tyrosine-protein phosphatase kappa | 3 |
| R-PTP-N2 | Receptor-type tyrosine-protein phosphatase N2 | 2 |
| R-PTP-O | Receptor-type tyrosine-protein phosphatase O | 2 |
| R-PTP-T | Receptor-type tyrosine-protein phosphatase T | 2 |
| SAP-97 | Disks large homolog 1 | 2 |
| SH2 domain protein C1 | SHC-transforming protein 1 | 2 |
| Sialyl Lewis x | Sialyl Lewis x | 1 |
| SKB1Hs | Protein arginine N-methyltransferase 5 | 2 |
| Sox11A | Transcription factor SOX-11 | 1 |
| STAP-1 | Signal-transducing adaptor protein 1 | 2 |
| STAP-2 | Signal-transducing adaptor protein 2 | 2 |
| STAT1 | Signal transducer and activator of transcription 1-alpha/beta | 2 |
| Surface Ag X | Surface Ag X | 1 |
| TACIP1 | Alpha-1-syntrophin | 2 |
| TBC1D9 | TBC1 domain family member 9 | 3 |
| TENS4 | Tensin-4 | 1 |
| TGF-β1 | Transforming growth factor beta-1 | 3 |
| TM peptide | Transmembrane peptide | 1 |
| TNFRSF14 | Tumor necrosis factor receptor superfamily member 14 | 2 |
| TNFRSF3 | Tumor necrosis factor receptor superfamily member 3 | 2 |
| TNF-α | Tumor necrosis factor | 3 |
| TNF-β* | Lymphotoxin-alpha | 4 |
| TopBP1 | DNA topoisomerase 2-binding protein 1 | 2 |
| UBC9 | SUMO-conjugating enzyme UBC9 | 2 |
| UBE2C | Ubiquitin-conjugating enzyme E2 C | 2 |
| UBP7 | Ubiquitin carboxyl-terminal hydrolase 7 | 2 |
| UCHL5 | Ubiquitin carboxyl-terminal hydrolase isozyme L5 | 1 |
| UPF3B | Regulator of nonsense transcripts 3B | 2 |
| VEGF* | Vascular endothelial growth factor | 4 |
| β-galactosidase | Beta-galactosidase | 1 |
