# Supplementary material for: Technical Advances of the Recombinant Antibody Microarray Technology Platform for Clinical Immunoproteomics
Source: PLoS One. 2016 Jul 14;11(7):e0159138. doi: 10.1371/journal.pone.0159138 (PMC4944972; doi:10.1371/journal.pone.0159138)
Supplement: S2 Table — To this end, microarray data for diseased group 3 vs. group 1 samples was used and compared with respect to No. of down-regulated scFvs antibodies and No. of complete matches per target molecule, using a fold change (FC) filter of FC > 1. (DOCX) [file pone.0159138.s007.docx]

**S2 Table.** **Evaluation of different normalization processes.** To this end, microarray data for diseased group 3 vs. group 1 samples was used and compared with respect to No. of down-regulated scFvs antibodies and No. of complete matches per target molecule, using a fold change (FC) filter of FC > 1.

| Diseased group 3 vs. Diseased group 1 | Subtract by group mean + semi-global | ComBat + Semi-global | Global VSN+ Combat | Global LOESS + Combat |
| --- | --- | --- | --- | --- |
| No. of down-regulated scFvs at FC > 1 (n_total_=195) | 46 | 49 | 87 | 95 |
| No. of complete matches* per target molecule (n_total_=47) | 25 | 25 | 19 | 20 |

* Each protein analyte was targeted by 2-9 individual scFv clones. A complete match is defined as when all scFvs per target gave similar results with respect to up- vs. down regulations using a fold change (FC) filter of FC > 1.
