# Supplementary material for: Technical Advances of the Recombinant Antibody Microarray Technology Platform for Clinical Immunoproteomics
Source: PLoS One. 2016 Jul 14;11(7):e0159138. doi: 10.1371/journal.pone.0159138 (PMC4944972; doi:10.1371/journal.pone.0159138)
Supplement: S3 Table — (DOCX) [file pone.0159138.s008.docx]

| **Subtract by group mean + semi-global** | |  |  | **ComBat + Semi-global** |  |  | **Un-normalized, logged data** |  |
| --- | --- | --- | --- | --- | --- | --- | --- | --- |
| **Antibody clone** | **q-value** | |  | **Antibody clone** | **q-value** |  | **Antibody clone** | **q-value** |
| Clone-51 | 3,12E-13 | |  | Clone-51 | 1,33E-13 |  | Clone-1 | 5,44E-14 |
| Clone-6 | 3,04E-11 | |  | Clone-6 | 1,97E-11 |  | Clone-2 | 5,37E-13 |
| Clone-7 | 4,89E-11 | |  | Clone-7 | 3,01E-11 |  | Clone-3 | 5,37E-13 |
| Clone-17 | 2,32E-10 | |  | Clone-17 | 1,12E-10 |  | Clone-4 | 8,35E-13 |
| Clone-5 | 9,90E-10 | |  | Clone-5 | 4,93E-10 |  | Clone-5 | 1,29E-12 |
| Clone-8 | 3,39E-09 | |  | Clone-4 | 1,69E-09 |  | Clone-6 | 1,29E-12 |
| Clone-21 | 3,48E-09 | |  | Clone-8 | 1,69E-09 |  | Clone-7 | 1,29E-12 |
| Clone-4 | 3,82E-09 | |  | Clone-21 | 1,69E-09 |  | Clone-8 | 1,29E-12 |
| Clone-19 | 3,82E-09 | |  | Clone-19 | 2,37E-09 |  | Clone-9 | 2,67E-12 |
| Clone-14 | 7,82E-09 | |  | Clone-14 | 3,66E-09 |  | Clone-10 | 4,64E-12 |
| Clone-52 | 1,05E-08 | |  | Clone-1 | 1,03E-08 |  | Clone-11 | 4,64E-12 |
| Clone-53 | 1,18E-08 | |  | Clone-34 | 1,03E-08 |  | Clone-12 | 2,68E-11 |
| Clone-34 | 1,28E-08 | |  | Clone-52 | 1,08E-08 |  | Clone-13 | 3,30E-11 |
| Clone-1 | 2,17E-08 | |  | Clone-10 | 1,08E-08 |  | Clone-14 | 3,55E-11 |
| Clone-10 | 2,17E-08 | |  | Clone-53 | 1,08E-08 |  | Clone-15 | 3,63E-11 |
| Clone-15 | 2,17E-08 | |  | Clone-15 | 1,10E-08 |  | Clone-16 | 3,91E-11 |
| Clone-54 | 2,63E-08 | |  | Clone-11 | 1,42E-08 |  | Clone-17 | 5,48E-11 |
| Clone-11 | 2,63E-08 | |  | Clone-41 | 1,71E-08 |  | Clone-18 | 5,48E-11 |
| Clone-41 | 2,95E-08 | |  | Clone-16 | 2,41E-08 |  | Clone-19 | 6,33E-11 |
| Clone-16 | 3,34E-08 | |  | Clone-38 | 2,77E-08 |  | Clone-20 | 1,07E-10 |
| Clone-55 | 3,82E-08 | |  | Clone-55 | 3,37E-08 |  | Clone-21 | 1,07E-10 |
| Clone-43 | 3,92E-08 | |  | Clone-43 | 3,37E-08 |  | Clone-22 | 1,14E-10 |
| Clone-38 | 3,92E-08 | |  | Clone-40 | 3,37E-08 |  | Clone-23 | 1,14E-10 |
| Clone-40 | 3,92E-08 | |  | Clone-54 | 3,57E-08 |  | Clone-24 | 1,29E-10 |
| Clone-24 | 5,98E-08 | |  | Clone-3 | 3,57E-08 |  | Clone-25 | 1,72E-10 |
| Clone-56 | 6,42E-08 | |  | Clone-24 | 3,57E-08 |  | Clone-26 | 1,72E-10 |
| Clone-28 | 6,82E-08 | |  | Clone-28 | 4,57E-08 |  | Clone-27 | 1,90E-10 |
| Clone-3 | 7,24E-08 | |  | Clone-36 | 4,57E-08 |  | Clone-28 | 2,18E-10 |
| Clone-36 | 8,29E-08 | |  | Clone-27 | 4,62E-08 |  | Clone-29 | 2,53E-10 |
| Clone-27 | 8,29E-08 | |  | Clone-56 | 4,95E-08 |  | Clone-30 | 2,85E-10 |
| Clone-20 | 8,77E-08 | |  | Clone-31 | 5,28E-08 |  | Clone-31 | 3,45E-10 |
| Clone-18 | 8,77E-08 | |  | Clone-29 | 5,41E-08 |  | Clone-33 | 4,27E-10 |
| Clone-29 | 8,77E-08 | |  | Clone-20 | 5,53E-08 |  | Clone-34 | 5,57E-10 |
| Clone-31 | 9,82E-08 | |  | Clone-18 | 6,07E-08 |  | Clone-35 | 6,97E-10 |
| Clone-57 | 9,87E-08 | |  | Clone-42 | 6,07E-08 |  | Clone-36 | 7,65E-10 |
| Clone-58 | 1,19E-07 | |  | Clone-57 | 9,56E-08 |  | Clone-37 | 1,19E-09 |
| Clone-42 | 1,24E-07 | |  | Clone-59 | 9,95E-08 |  | Clone-38 | 1,38E-09 |
| Clone-59 | 1,35E-07 | |  | Clone-58 | 1,26E-07 |  | Clone-39 | 1,85E-09 |
| Clone-32 | 1,87E-07 | |  | Clone-32 | 1,26E-07 |  | Clone-40 | 2,14E-09 |
| Clone-60 | 2,07E-07 | |  | Clone-60 | 1,48E-07 |  | Clone-41 | 2,33E-09 |
| Clone-61 | 2,21E-07 | |  | Clone-46 | 2,25E-07 |  | Clone-42 | 2,51E-09 |
| Clone-62 | 2,68E-07 | |  | Clone-62 | 2,25E-07 |  | Clone-43 | 2,87E-09 |
| Clone-46 | 2,94E-07 | |  | Clone-61 | 2,41E-07 |  | Clone-44 | 3,00E-09 |
| Clone-63 | 2,99E-07 | |  | Clone-13 | 3,00E-07 |  | Clone-45 | 3,07E-09 |
| Clone-64 | 2,99E-07 | |  | Clone-65 | 3,23E-07 |  | Clone-46 | 3,20E-09 |
| Clone-65 | 3,05E-07 | |  | Clone-69 | 3,23E-07 |  | Clone-47 | 3,24E-09 |
| Clone-69 | 3,05E-07 | |  | Clone-64 | 3,23E-07 |  | Clone-48 | 3,41E-09 |
| Clone-67 | 4,05E-07 | |  | Clone-30 | 3,28E-07 |  | Clone-49 | 3,95E-09 |
| Clone-30 | 4,47E-07 | |  | Clone-70 | 3,80E-07 |  | Clone-50 | 5,41E-09 |
| Clone-68 | 4,82E-07 | |  | Clone-67 | 3,90E-07 |  | Clone-32 | 5,56E-09 |

**S3 Table. Top 50 differentially expressed proteins (represented by their matching antibody clone) for diseased vs healthy controls, before and after normalization.**
